# Bladder mucosal afferents detect UTI and aid pathogen clearance

**DOI:** 10.1073/pnas.2604124123

**Published:** 2026-08-03

**Authors:** Cindy Tay, Harman Sharma, Stewart Ramsay, Georgia Bourlotos, Sarah K. Manning, Natalie E. Stevens, Sophie J. Miller, Geraint B. Rogers, David J. Lynn, Feargal J. Ryan, Andrea M. Harrington, Vladimir Zagorodnyuk, Steven L. Taylor, Luke Grundy

**Affiliations:** ^a^ College of Medicine and Public Health, Flinders Health and Medical Research Institute, Flinders University, Bedford Park, SA 5042, Australia; ^b^ Lifelong Health, South Australian Health and Medical Research Institute, Adelaide, SA 5000, Australia; ^c^Adelaide Medical School, The University of Adelaide, Adelaide, SA 5000, Australia; ^d^ Microbiome and Host Health Programme, South Australian Health and Medical Research Institute, Adelaide, SA 5000, Australia; ^e^ Precision Cancer Medicine Theme, South Australian Health and Medical Research Institute, Adelaide, SA 5000, Australia; ^f^ Visceral Pain Research Group, Hopwood Centre for Neurobiology, Lifelong Health Theme, South Australian Health and Medical Research Institute, Adelaide, SA 5000, Australia; ^g^School of Biomedicine, Faculty of Health and Medical Sciences, University of Adelaide, Adelaide, SA 5005, Australia

**Keywords:** urinary tract infection, bladder afferent, cystitis, pain, infection

## Abstract

The bladder is innervated by a complex network of sensory neurons that detect and transmit mechanical and chemical signals to the central nervous system, regulating both urine storage and voiding, and mediating sensations of bladder fullness and pain. While stretch-sensitive afferents within the bladder wall are known to monitor bladder tension, the contribution of stretch-insensitive afferents that reside within the bladder mucosa to bladder sensation and function remains unclear. Here, we establish a mouse model of selective bladder mucosal afferent denervation using intravesical instillation of resiniferatoxin to define the functional roles of these fibers in health and disease. We assessed the impact of mucosal denervation on sensory nerve activity and bladder function in healthy mice and in a model of urinary tract infection (UTI) induced by acute bacterial challenge with uropathogenic *Escherichia coli*. We show that mucosa-innervating afferents do not influence bladder distension responses or voiding function under healthy conditions. In contrast, during UTI, these afferents become hypersensitive and serve as important drivers of infection-induced pelvic pain and urinary frequency, responses that contribute to bacterial clearance. Together, these findings identify bladder mucosal afferents as key sensors of pathogenic challenge and reveal a previously unrecognized mechanism that drives behavioral responses to reduce the burden and extent of UTI.

## Significance

The bladder is innervated by complex sensory networks, yet the specific role of stretch-insensitive mucosal nerves has remained unknown for nearly two decades. Utilizing a model of selective mucosal afferent denervation we demonstrate that these fibers are dispensable for normal bladder function, but are essential for detecting urinary tract infections (UTIs). We show that mucosal afferents are important contributors of UTI-induced bladder pain and increased urinary frequency, a behavioral response that actively facilitates pathogen clearance. Without these sensory cues, bacterial burden increases and the risk of UTI dissemination to the kidneys rises. These findings identify bladder afferents as a key component of the host’s innate defense against infection.

The bladder is innervated by a complex network of sensory nerves with functionally diverse properties that allow the detection of both mechanical and chemical stimuli (1–5). Bladder-innervating afferents have cell bodies within the dorsal root ganglia (DRG) and terminate within the dorsal horn of the spinal cord where they feed into the central nervous system circuits responsible for regulating bladder sensation and function (6–8). Around 75 to 80% of all bladder afferent endings are found within the detrusor smooth muscle where they are responsible for detecting the degree of bladder wall stretch (4, 5, 9) and transducing sensory signals to the brain to elicit sensations of bladder fullness necessary to support normal bladder function and maintain homeostasis (4, 6, 7, 10). The remaining 20 to 25% of afferents innervating the bladder have endings closer to the bladder lumen, within the bladder mucosa, and a significant proportion of these exhibit no response to physiological levels of bladder stretch (4, 5, 11–13). The role of these stretch-insensitive mucosal afferents in bladder function has yet to be determined. However, their greater chemosensitivity (4, 12, 14) and location within the superficial layers of the bladder where infection and inflammation predominate (15), has led us to hypothesize that they may serve a crucial role in detecting and signaling the presence of pathological stimuli.

Urinary tract infections (UTIs) are one of the world’s most prevalent infections and the most common pathophysiological challenge to the bladder (16). UTIs are characterized by the development of irritative and painful sensations that are highly distinct from those experienced during healthy bladder function, including pelvic pressure/pain, dysuria (pain/burning sensation when urinating), and severe urgency that drive an increase in urinary frequency (1, 2, 8, 17). In addition to alerting the host to the presence of bladder pathophysiology, these symptoms have also been proposed as important regulators of pathogen clearance by driving an increase in the frequency of urination (8, 18, 19). However, it remains unclear if specific subtypes of bladder afferents are responsible for signaling distinct aspects of bladder sensation to drive these sensory and behavioral responses.

Here we address this knowledge gap by developing a mouse model of bladder mucosal afferent denervation utilizing the neurotoxin resiniferatoxin (RTX) and assessing the consequences on sensory nerve fiber activity, bladder function, and bacterial persistence during acute bacterial challenge. Our results implicate mucosal afferents as important contributors to the detection of pathological challenge in the bladder and the initiation of urinary frequency to reduce the burden and extent of acute bladder infection.

## Results

### Intrabladder Resiniferatoxin Selectively Denervates Mucosal Afferents.

**Fig. 1.**
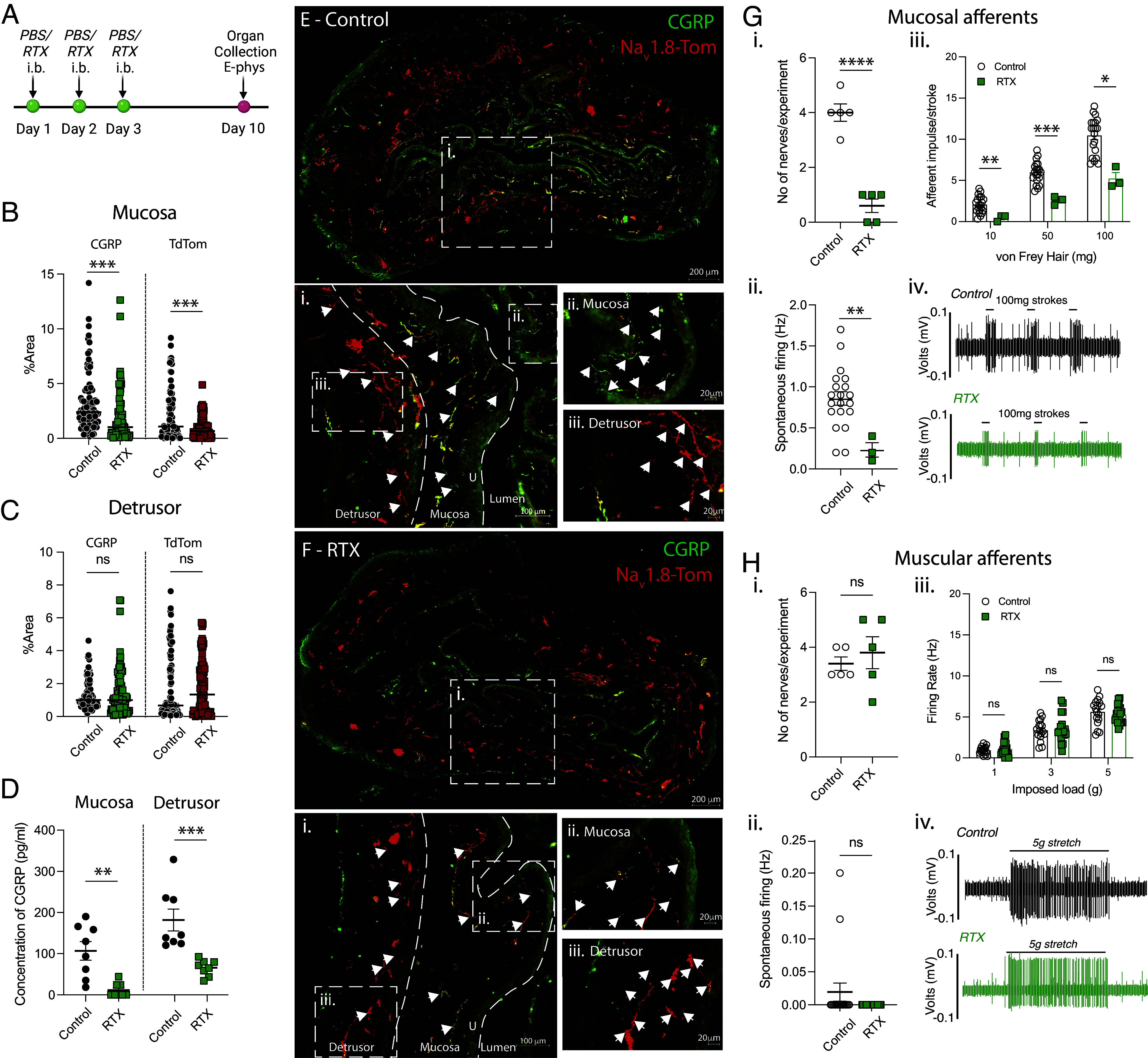
Intravesical RTX reduces the density of CGRP-immunoreactive and Nav1.8-tdtomato expressing fibers in the bladder mucosa and reduces mechano-sensitivity of mucosa innervating afferents. (*A*) Schematic representation of the experimental design for the murine mucosal denervation model. Mice received either RTX or PBS (SHAM) for three consecutive days and bladders were assessed 7 d after the final infusion. (*B* and *C*) Quantitative data from cross-sections of bladders showing % Area of the mucosa (*B*) and the detrusor (*C*) covered by Nav1.8-TdTom and CGRP-AF488 fluorescence (n = 90 sections from N = 5 mice/experimental group). (*D*) Quantification of the protein concentration of CGRP between control and RTX treated bladders (N = 8/experimental group). Representative photomicrographs showing the distribution of CGRP-immunoreactive fibers (green) and Na_v_1.8-tdTomato expressing fibers (red) in cross-sections (40 µm) of the bladder of control (*E*) and RTX-treated (*F*) mice. (*E* and *F*, *i*) High magnification of the bladder wall rotated 90° from whole bladder sections showing CGRP-immunoreactive and Na_v_1.8-tdTomato expressing fibers innervating the detrusor, lamina propria, and urothelium (Mucosa). *Insets* from (*E* and *F*, *i*) show CGRP-immunoreactive and Na_v_1.8-tdTomato expressing fibers within the mucosa (*E* and *F*, *ii*), and detrusor (*E* and *F*, *iii*) at higher magnification. Mucosal afferents. (*G*, *i*) The number of functional mucosal afferents identified from each independent experiment (N = 5/group). (*G*, *ii*) Mucosal spontaneous firing frequency (n = 20-3 responsive units from N = 5 mice/group). (*G*, *iii*) Mucosal afferent responses to von Frey Hair stroking (10, 50, 100 mg) in remaining mucosal afferents from N = 5 mice. (*G*, *iv*) Representative mucosal afferent nerve profiles of control (black) and RTX (green) treated bladders to 100 mg mucosal stroking. (*H*) Muscular afferents. (*H*, *i*) The number of muscular afferents identified from individual experiments (N = 5). (*H*, *ii*) Spontaneous firing frequency of muscular afferents (n = 17 to 19 responsive units from N = 5 mice/group). (*H*, *iii*) Muscular afferent responses to bladder stretch by imposed load (1, 3, 5 g) and (*H*, *iv*) Representative response profiles of i) control (black) and ii) RTX (green) treated bladders to 5 g bladder stretch (N = 5 mice). Data presented as mean ± SEM. ^ns^*P* > 0.05, *P < 0.05, **P < 0.01, ***P < 0.001, and ****P < 0.0001. Statistical differences for *B* and *C* determined using the nonparametric Mann–Whitney test. Data in *D* analyzed by Welch’s *t* test. *G* and *H*, *i* and *ii*, were determined by the unpaired Mann–Whitney test. For *G* and *H*, *iii*, statistical differences were determined by two-way ANOVA with Geisser–Greenhouse correction and Šídák multiple comparisons.

Understanding the contribution of mucosal afferents to bladder sensation and function has been hindered by a lack of in vivo models to effectively and specifically isolate their function. To address this challenge, we leveraged the potent neurodegenerative properties of resiniferatoxin (RTX) (20–22) with the known expression of TRPV1 within bladder afferents (23, 24), and the ability to instill compounds within the bladder lumen following urethral catheterization. Combined with the known barrier role of the urothelium, biological diffusion properties, and exposure time to restrict the toxins reach, these conditions allowed us to selectively denervate TRPV1-expressing neurons in the bladder mucosa (Fig. 1*A*).

Throughout the bladder, we identified dense sensory networks in the detrusor and mucosa (urothelium and lamina propria) via endogenous tdTomato fluorescence from our Nav1.8-tdTom mice and CGRP immunoreactivity (Fig. 1 *B*, *C*, *E*, and *F* and *SI Appendix*, Figs. S1–S3). Seven days after intravesical RTX instillation, we observed a markedly reduced density in these innervation markers in the bladder mucosa (Fig. 1*B* and Fig. 1 *E* and *F*, *ii*), but not in the detrusor (Fig. 1*C* and Fig. 1 *E* and *F*, *iii*) indicating selective denervation of mucosal afferents. Analysis of distinct regions of the bladder wall (Neck, Middle, Dome) revealed consistent and specific loss of CGRP fluorescence in the mucosa (*SI Appendix*, Figs. S1 and S2). Quantification of CGRP concentration within excised mucosa and detrusor tissue showed almost complete abolishment of CGRP in the mucosa, and a significant decrease in CGRP within the detrusor (Fig. 1*D*). The effect of RTX on TdTom^+^ fluorescence was greatest within the mucosa of the bladder dome (*SI Appendix*, Figs. S1 and S3). Importantly, loss of mucosal afferents 7 d after RTX instillations occurred in the absence of obvious changes in bladder structure (*SI Appendix*, Fig. S4), or resident immune cell populations (*SI Appendix*, Fig. S5), confirming the specificity of RTX in targeting sensory innervation and rapid recovery of any potential neurogenic inflammation induced by RTX treatments (25).

The functional impacts of intravesical instillation of RTX on bladder sensory signaling were assessed through changes in mechanosensory responses of the major bladder afferent subtypes including mucosal (Fig. 1*G*), muscular (Fig. 1*H*), and muscular-mucosal (*SI Appendix*, Fig. S6), afferents (4, 5). Aligning with our anatomical data, mucosal afferents, which do not respond to bladder stretch but can be activated by light urothelial stroking (4, 5), were mostly absent from RTX-treated electrophysiology preparations (Fig. 1 *G*, *i*). Of the mucosal afferents that retained activity, both spontaneous firing frequency (Fig. 1 *G*, *ii*) and mechanosensory responses to stroking were significantly reduced compared to sham preparations (Fig. 1 *G*, *iii* and *iv*). The number of functionally responsive muscular-mucosal afferents, which respond to both mucosal stroking and bladder stretch and have endings that extend throughout the mucosa and detrusor, were also significantly reduced following RTX treatment (*SI Appendix*, Fig. S6, *i*). RTX-treatment had no significant impact on spontaneous firing frequency (*SI Appendix*, Fig. S6, *ii*) or mechanosensory responses to either mucosal stroking (*SI Appendix*, Fig. S6, *iii* and *iv*) or bladder stretch (*SI Appendix*, Fig. S6, *v* and *vi*) of remaining muscular-mucosal afferents. In contrast to effects on mucosal and muscular-mucosal afferents, RTX treatment had no significant impact on the number of muscular afferents per preparation (Fig. 1 *H*, *i*), spontaneous firing frequency (Fig. 1 *H*, *ii*), or mechanosensory responses to both low- and high- intensity load (1 to 5 g) induced stretch (Fig. 1 *H*, *iii* and *iv*). Taken together these data indicate that RTX treatment potently and specifically reduces innervation and function of afferents with endings in the bladder mucosa without major impacts on stretch-sensitive muscular afferents innervating the detrusor smooth muscle.

### Denervation of Mucosal Afferents Has No Impact on Normal Bladder Sensation and Function.

**Fig. 2.**
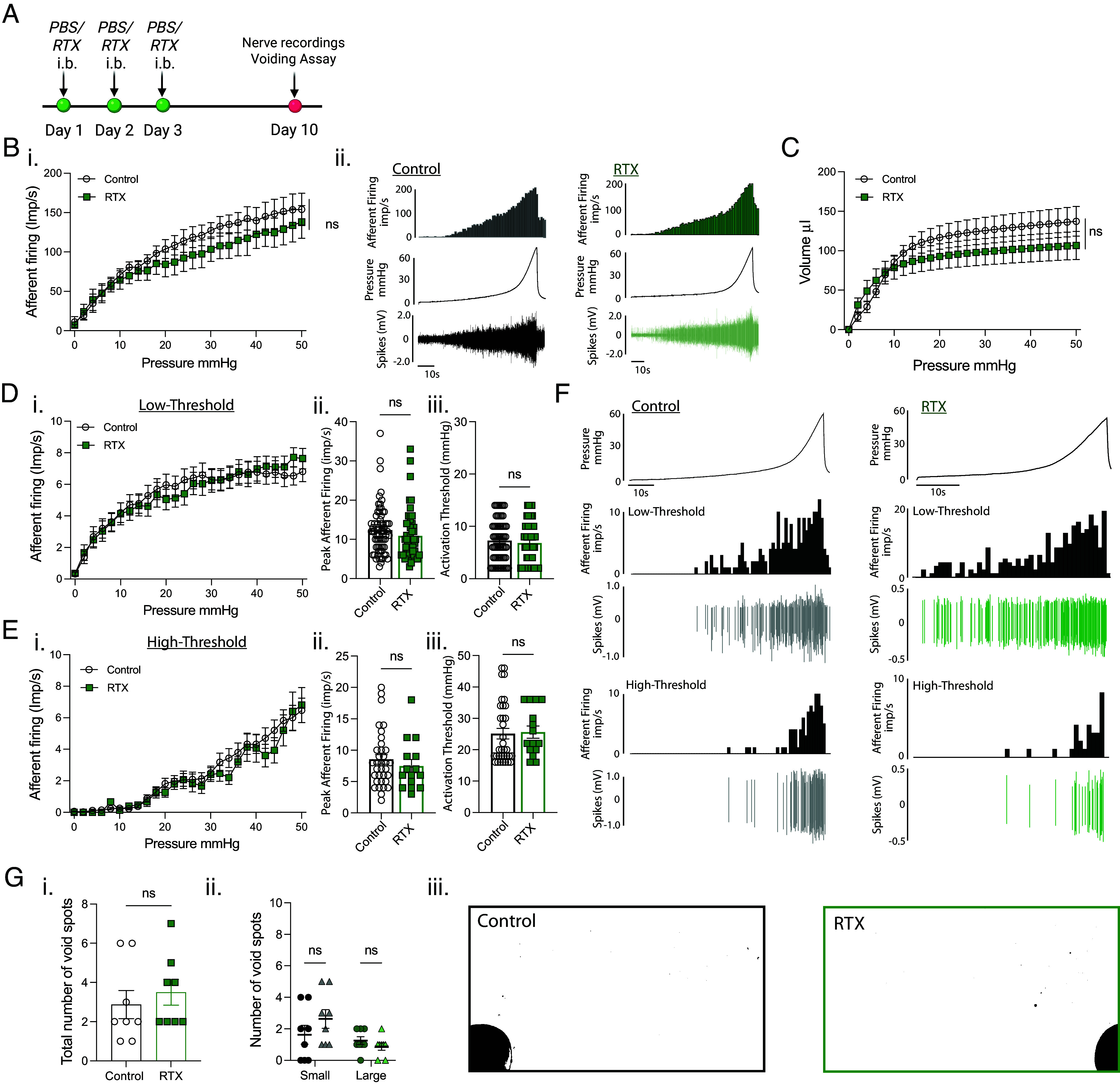
Mucosal afferent denervation does not impact bladder afferent mechanosensitive responses to distension ex vivo or in vivo bladder voiding patterns. (*A*) Schematic representation of the experimental design. Mice received either RTX or PBS (SHAM) for three consecutive days and bladders were assessed 7 d after the final infusion. (*B*) Ex vivo bladder afferent sensitivity to graded distension (0 to 50 mmHg) in control- and RTX-treated mice (*B*, *i*) (N = 5 mice). (*B*, *ii*) Representative ex-vivo bladder afferent experimental trace showing intravesical pressure (mmHg), raw spike activity (mV), and afferent firing (imp/s) in response to graded bladder distensions in control (black) and RTX-treated (green) bladders. (*C*) Bladder muscle pressure/volume relationship (N = 5 mice). Single units were extracted from multiunit bladder afferent recording experiments and separated based on their activation threshold to distension as either low- (*D*), or high-threshold (*E*). (*D*, *i*) Low-threshold bladder afferent responses to distension; (*E*, *ii*) maximum firing rate during distension; (*D*, *iii*) and activation threshold (mmHg) (n = 52 to 69 individual units from N = 5). (*E*, *i*) High-threshold bladder afferent responses to distension; (*E*, *ii*) maximum firing rate during distension; (*E*, *iii*) and activation threshold (mmHg) (n = 15 to 34 individual units from N = 5). (*F*) Representative experimental traces showing mechanosensitivity of low-threshold and high-threshold afferent units in response to graded bladder distensions (0 to 50 mmHg) from control (black) and RTX (green) treated bladders. (*G*) Analysis of voiding behavior in control and RTX-treated mice including total void spots (N = 8 mice) (*G*, *i*), and number of small and large sized void spots (*G*, *ii*) from control and RTX treated mice (N = 8 mice). (*G*, *iii*) Representative images of voiding patterns in control and RTX treated mice. Data are presented as mean ± SEM. ^ns^*P* > 0.05. Individual data points represent values from individual afferent units. Statistical differences for data in *A*, *i*, *B*, *C*, *i*, and *D*, *i* were determined by Two-way ANOVA with Šídák multiple comparisons. Statistical differences for data in *C* and *D*, *ii* and *iii* were determined by the unpaired Mann–Whitney test. Statistical differences for data in Fii were determined by two-way ANOVA with Bonferroni’s multiple comparisons.

Awareness of bladder fullness is provided by integration of mechanosensory signals from the bladder into the central micturition circuits that regulate bladder filling and emptying and is essential for normal physiological functioning of the bladder (6–8). Our RTX-model of mucosal denervation provided a unique opportunity to explore the contribution of mucosal afferents to bladder sensory signaling during bladder filling in an intact bladder (Fig. 2 *A*–*D*). Using an ex vivo whole bladder afferent recording preparation we observed robust afferent responses to graded bladder distension (0 to 50 mmHg) in both RTX and sham treated bladders (Fig. 2 *B*, *i* and *ii*), with no significant difference in afferent response to distension (Fig. 2 *B*, *i* and *ii*) and no change in bladder compliance (Fig. 2*C*) following RTX treatment. Subsequent analysis of single mechanosensory units confirmed no difference in firing frequency or activation threshold in response to distension between bladder afferents from control and RTX treated mice (*SI Appendix*, Fig. S7), nor any differences in the firing patterns of low- and high-threshold subtypes (Fig. 2 *D* and *E*) including peak afferent response (Fig. 2 *D* and *E*, *ii*), activation threshold (Fig. 2 *D* and *E*, *iii*), or area under the curve (AUC) (*SI Appendix*, Fig. S8). Aligning with the lack of effect of RTX on mechanosensory afferent responses or changes in bladder compliance, RTX had no impact on in vivo bladder function (Fig. 2*G*). Quantification of in vivo bladder voiding behavior found no difference in the number of void spots (Fig. 2 *G*, *i*–*iii*), and no differences in the proportion of small or large void spots (Fig. 2 *G*, *ii* and *iii*) between control and RTX-treated mice. Together these findings indicate that mucosal afferents are not significant mediators of bladder distension-induced mechanosensory responses and bladder function under normal physiological conditions.

### Denervation of Mucosal Afferents Impacts Bladder Sensation and Function During UTI and Increases Bacterial Burden and Inflammation.

**Fig. 3.**
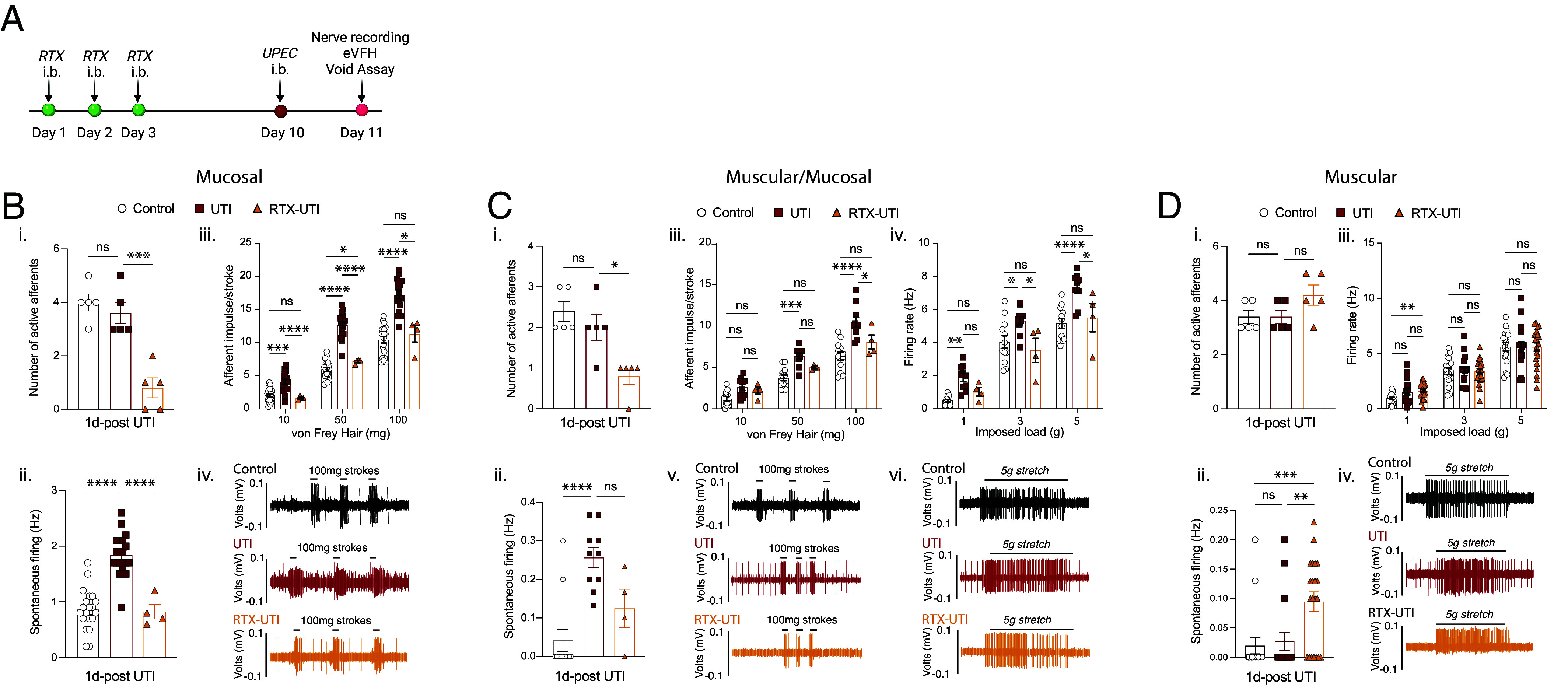
Mucosal but not muscular afferents are sensitized during UTI. (*A*) Schematic representation of the experimental design for the combined murine mucosal denervation and UTI infection model. (*B*) Mucosal afferents: (*B*, *i*) The number of mucosal afferents identified from individual experiments [control (black) n = 20, UTI (red) n = 18, RTX-UTI (orange) n = 4, from N = 5 mice]. (*A*, *ii*) Spontaneous firing frequency of mucosal afferents. (*B*, *iii*) Mucosal afferent responses to von Frey Hair stroking (10, 50, 100 mg). (*A*, *iv*) Representative mucosal afferent nerve recording traces from control (black), UTI (red), and RTX-UTI (orange) treated bladders in response to 100 mg mucosal stroking. (*C*) Muscular-Mucosal afferents: (*C*, *i*) The number of mucosal nerves identified from individual experiments [control (black) n = 12, UTI (red) n = 10, RTX-UTI (orange) n = 4, from N = 5 mice]. (*C*, *ii*) Spontaneous firing frequency of muscular-mucosal afferents. (*C*, *iii*) Muscular-mucosal afferent responses to von Frey Hair stroking (10, 50, 100 mg). (*C*, *iv*) Muscular-mucosal afferent responses to bladder stretch by imposed load (1, 3, 5 g). Representative bladder afferent nerve recording profiles of control (black), UTI (red), and RTX-UTI (orange) treated bladders to 100 mg mucosal stroking (*C*, *v*) and 5 g bladder stretch (Cvi). (*D*) Muscular afferents: (*D*, *i*) The number of muscular nerves identified from individual experiments [control (black) n = 17, UTI (red) n = 17, RTX-UTI (orange) n = 21, from N = 5 mice]. (*D*, *ii*) Spontaneous firing frequency of muscular afferents. (*D*, *iii*) Muscular afferent responses to bladder stretch by imposed load (1, 3, 5 g). (*D*, *iv*) Representative muscular afferent nerve recording traces from control (black), UTI (red), and RTX-UTI (orange) treated bladders in response to 5 g stretch. Data are presented as mean ± SEM. ^ns^*P* > 0.05, *P < 0.05, **P < 0.01, ***P < 0.001, and ****P < 0.0001. Statistical differences for *A*–*C*, *i* were determined by the Kruskal–Wallis test with Dunn’s multiple comparisons. For *A*–*C*, *ii*, statistical differences were determined by Brown–Forsythe and Welch ANOVA test with Dunnett T3 multiple comparisons. For *A*, *iii*, *B*, *iii* and *v*, and *C*, *iii* statistical differences were determined by two-way ANOVA with Geisser–Greenhouse correction and Tukey’s multiple comparisons.

Despite their limited role in bladder function under healthy conditions, the dense sensory innervation of the mucosa (Fig. 1 and *SI Appendix*, Fig. S1) (9, 11) hints at a critical role in bladder homeostasis. Positioned near the bladder lumen, mucosal afferents are uniquely situated to rapidly detect infection or inflammation where it predominates during UTI (26, 27). Given that infection represents a significant evolutionary pressure, with early detection critical for host survival (28), we hypothesized that mucosal afferents may exist to detect pathological stimuli such as UTIs. To test this, we combined an established mouse model of UTI (1, 3) with our intravesical RTX model to selectively denervate mucosal afferents and evaluate their role in infection detection. UTI was induced 7 d after RTX treatment (Fig. 3*A*); a timepoint at which there were significant reductions in mucosal afferent innervation and function (Figs. 1 and 2) but there were no differences in bladder structure (*SI Appendix*, Fig. S4) or resident immune cell populations (*SI Appendix*, Fig. S5), providing a stable physiological baseline free from potential confounding effects of acute ablation.

Twenty-four hours after bladder inoculation with uropathogenic *Escherichia coli* (UPEC) strain CFT073, mice developed acute UTI with bacteria detectable within the urine (*SI Appendix*, Fig. S9), a robust immune response characterized by neutrophil and inflammatory monocyte infiltration (15), and significant bladder damage and edema that predominates in the urothelium and lamina propria (15, 26, 27) (*SI Appendix*, Fig. S10). Ex vivo bladder afferent nerve recordings revealed no changes in the number of mucosal afferents identified per experiment (Fig. 3 *B*, *i*). However, mucosal afferents exhibited significant increases in the frequency of spontaneous firing (Fig. 3 *B*, *ii*) and significant hypersensitivity to mucosal stroking 24 h after UPEC infusion compared to noninfected mice (Fig. 3 *B*, *iii* and *iv*). Additionally, muscular-mucosal afferents exhibited no changes in the number of mucosal afferents identified per experiment (Fig. 3 *C*, *ii*) but showed increased spontaneous firing (Fig. 3 *C*, *ii*) and developed significant hypersensitivity to both mucosal stroking (Fig. 3 *C*, *iii* and *v*) and stretch during UTI when compared to bladders from sham treated mice (Fig. 3 *C*, *iv* and *vi*). In contrast, muscular afferents showed no differences in spontaneous firing (Fig. 3 *D*, *ii*) or responses to bladder stretch following UPEC instillation (Fig. 3 *D*, *iii* and *iv*). Intravesical RTX treatment prior to UPEC instillation (RTX-UTI) significantly reduced the number of active mucosal and muscular mucosal afferents (Fig. 3 *B* and *C*, *i*), and prevented UTI-induced sensitization of mucosal (Fig. 3 *B*, *ii*–*iv*) and muscular-mucosal afferent mechanosensory responses (Fig. 3 *C*, *ii*–*vi*). Mucosal denervation with RTX prior to UTI did not reduce the number of muscular afferents (Fig. 3 *D*, *i*) but induced a significant increase in spontaneous firing of muscular afferents during UTI without changing their mechanosensitivity (Fig. 3*D*). Together these data implicate mucosal afferents as specific drivers of sensory signaling from the bladder during UTI.

**Fig. 4.**
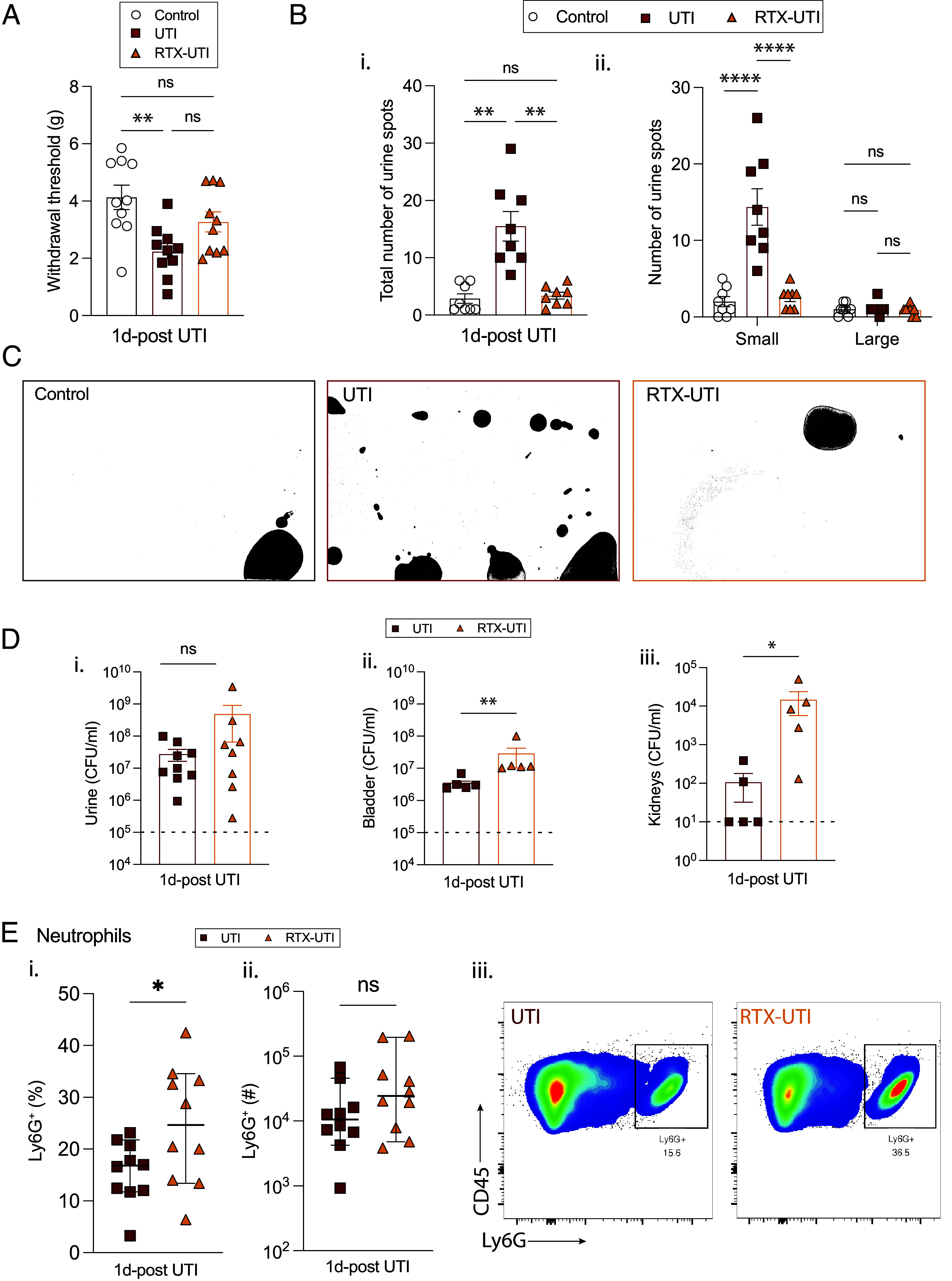
Mucosal afferents regulate bladder host-defense responses. (*A*) Withdrawal thresholds (g) to eVFH stimulation (N = 10). (*B*) Analysis of voiding behavior including total void spots (*B*, *i*), and number of small and large sized void spots (*B*, *ii*) from control, RTX, and RTX-UTI treated mice (N = 8). (*C*) Representative images of voiding patterns. (*D*) Bacterial load in bladder (*D*, *i*), urine (*D*, *ii*), and kidneys (*D*, *iii*) 24 h after mice were infected with UPEC (N = 5 to 10 mice). The dotted line represents the limit of detection for urine (10^5^), bladder (10^2^), kidneys (10^1^). (*E*) Neutrophil frequency as a percentage of total viable bladder immune (CD45^+^) cells (*E*, *i*) and total bladder neutrophil counts (*E*, *ii*) in UTI and RTX-UTI mice (N = 10 mice). (*E*, *iii*) Representative flow cytometry plots of Ly6G^+^ neutrophils from UTI and RTX-UTI mice 24 h after UPEC infection. Data are presented as mean ± SEM, ^ns^*P* > 0.05, *P < 0.05, **P < 0.01, ****P < 0.0001. Individual data points represent values from a single mouse. Statistical differences for *A* and *B* were determined by Brown–Forsythe and Welch ANOVA tests with Dunnett T3 multiple comparisons. For *D* statistical differences were determined by the unpaired *t* test. Data in *E*, *i* analyzed by Welch’s *t* test and *E*, *ii* by the unpaired Mann–Whitney test.

In humans, UTIs are typically associated with the development of distinct bladder sensations, including urinary urgency, dysuria, and pelvic pain which drive protective behaviors including an increase in the frequency of urination that can be recreated in mouse models (1, 2, 6–8, 17). To determine the consequences of inhibiting mucosal sensory signaling during UTI on the development of these protective behavioral responses, we assessed the development of pelvic pain and bladder dysfunction in UTI and RTX-UTI treated mice (Fig. 4). Our data show that 24 h after UPEC instillation, mice developed pelvic hyperalgesia (Fig. 4*A*) and urinary frequency (Fig. 4 *B*, *i*), characterized by increased numbers of small urine spots during voiding analysis (Fig. 4 *B*, *ii* and Fig. 4*C*). In contrast, intravesical RTX treatment prior to UPEC instillation mitigated the development of pelvic hyperalgesia (Fig. 4*A*) and prevented the development of urinary frequency (Fig. 4 *B*, *i* and *ii* and Fig. 4*C*).

Behavioral responses to UTI are considered important in infection clearance by provoking more rapid excretion of UPEC growing in the urine, along with exfoliated infected urothelial cells (15). To explore the consequences of inhibiting this host response on bacterial clearance, we compared the burden and extent of UTI following UPEC instillation in control UTI and RTX treated (RTX-UTI) mice (Fig. 4*D*). Given the variable characteristics of UPEC strains, experiments were performed using UPEC strain CFT073 (Fig. 4*D*) and UTI89 (*SI Appendix*, Fig. S11). While urine levels of UPEC CFT073 were elevated but not significant (Fig. 4 *D*, *i*), levels in tissue of the bladder wall and kidneys were significantly higher (Fig. 4 *D*, *ii* and *iii*) in RTX-treated mice. Notably, a greater proportion (100% versus 40% in control UTI) of mice showed infection ascended to the kidneys following mucosal denervation with RTX (Fig. 4 *D*, *iii*). Similar findings were identified using UPEC UTI89, with significantly higher bacteria levels in urine and bladder tissue of RTX-UTI mice (*SI Appendix*, Fig. S11 *A* and *B*), while kidney CFU were elevated but not significant (*SI Appendix*, Fig. S11*C*).

The host-defense response to UTI includes a substantial innate immune response that is necessary for infection clearance (15, 26, 27). To assess whether mucosal denervation with RTX changed this response we utilized flow cytometry to assess the frequency of major immune cell populations including T cells, monocytes, macrophages, neutrophils, and NK cells (Fig. 4 and *SI Appendix*, Fig. S12). The increase in UPEC burden seen in the bladders of RTX-UTI mice was associated with a higher relative abundance of neutrophils (Fig. 4 *E*, *i* and *iii*) without significant changes in the frequency or number of other immune cell populations (*SI Appendix*, Fig. S12).

## Discussion

In health, bladder sensation primarily reflects the degree of bladder fullness, gradually intensifying as the bladder fills until voiding is initiated (7, 8). During a UTI, however, the relationship between bladder volume and sensation becomes dysregulated, with bladder sensations dominated by irritative and painful sensations, including pelvic pain/pressure, dysuria, and urgency even when the bladder is not full (8, 29). In this study, we present data to support a hypothesis whereby discrete functional subclasses of bladder afferents differentially contribute to sensation and bladder function in health versus disease. Using a mouse model of mucosal denervation, we demonstrate that muscular afferents govern bladder mechanosensation and voiding function in health while mucosal afferents emerge as the dominant drivers of sensory signaling during infection to initiate urinary frequency that contributes to the host-defense response to infection.

As normal function of the bladder is related to the accurate detection of bladder muscle stretch as a proxy for bladder fullness, the role of stretch-insensitive mucosal sensory nerves in bladder sensation and function has been an enduring question since their discovery almost 20 y ago (4, 5). Experimental interrogation of mucosal afferents has been constrained by two major limitations: first, there are no known molecular markers that reliably distinguish muscular and mucosal afferents, precluding their selective chemogenetic or cre-inducible ablation (22, 30, 31); and second, the absence of clear anatomical segregation by spinal level (5, 6) blunts the potential of recently developed techniques for targeted dorsal root ganglia (DRG) removal (32). RTX has long been used as a molecular scalpel to ablate TRPV1^+^ nerves via central or systemic administration (20, 22). However, because TRPV1 is widely expressed throughout bladder afferents (23, 24, 29), these approaches lack the spatial precision needed to study mucosal afferent function in isolation. To overcome this limitation, we exploited the barrier and diffusion properties of the urothelium to develop a model of spatially selective mucosal denervation by infusing RTX intravesically into the bladder lumen over three consecutive days. However, this method was not without limitations in its specificity, as we saw reductions in detrusor CGRP concentrations following RTX and altered spontaneous firing in muscular afferents in RTX-UTI mice that raise the possibility of broader sensory modulation. The reductions in CGRP in the absence of changes in muscular afferent function likely reflect the diffusion of RTX into the superficial detrusor at a concentration that could cause TRPV1 activation and neuropeptide depletion without neuronal death (33–35). The mechanism underlying an increase in spontaneous firing of muscular afferents are unknown, but one possibility is that the increased bacterial load in the bladder as a consequence of mucosal denervation by RTX (Fig. 4*D*) correlates with a greater depth of bacterial invasion into the bladder wall, providing greater opportunity for interactions between bacteria and host response mediators that could drive sensitization of some muscular afferents. Despite these limitations, our combined data show our approach produced a robust and specific attenuation of mucosal afferent signaling, and reductions in CGRP^+^ and TdTom^+^ mucosal innervation while preserving muscular afferent innervation and function. This model thus enabled a systematic dissection of the functional specialization of muscular and mucosal nerves. Using graded bladder distension as a proxy for normal bladder filling, and spontaneous voiding as an assessment of healthy bladder function, we found pelvic afferent responses and bladder function in healthy control mice were unchanged following mucosal denervation. In contrast, we show mucosal afferents are important for signaling the presence of UTI, inducing pelvic pain, and increasing urinary frequency.

Sensory nerves have a well-known role in detecting and signaling pathological stimuli from both the skin and viscera to elicit unique sensations and protective behavioral responses (28). Infection and inflammation are classical drivers of neuronal sensitization (25, 36), and both bacterial and inflammatory stimuli have been shown to sensitize bladder DRG (2, 3) and induce pelvic hyperalgesia and urinary frequency (1, 2, 17, 37). How mucosal innervating afferents are exclusively sensitized during UTI, however, remains unclear. It is possible that mucosal afferents exhibit a unique phenotype that mediates responses to stimuli of distinct modalities through unique ion-channel/receptor profiles in line with the specificity theory of labeled lines (38). In support, muscular and mucosal afferents in mice have known differences in mechanosensitivity (4, 5), neuropeptide content (11, 39), and sensitivity to chemical stimuli (12–14) that hint at unique molecular profiles that may underpin a unique function. An alternative proposition is that the functional specificity of mucosal afferents we observed may be directly related to their location within the urothelium and lamina propria, where infection and inflammation predominate during UTI (15). Patients with clinically distinct forms of noninfectious cystitis, including interstitial, radio-, immuno-, and chemotherapy-induced cystitis typically describe bladder sensations and symptoms in line with those experienced during UTI (40–42), suggesting the same neural mechanisms may underlie these sensations and implicating mucosal afferents as broad sensors of pathological stimuli, including both infection and inflammation. Similarly, mucosal afferents are preferentially sensitized in a noninfectious guinea pig cystitis model, and cystitis induced nocifensive visceromotor responses to bladder distension are reduced following acute intravesical instillation of RTX (43). Mucosal afferents can also be sensitized or respond directly to a broad variety of inflammatory molecules (12–14); and undergo immune-mediated sprouting that drives pelvic sensitivity and urinary frequency after recurrent UTI (37). When spatial proximity is removed, such as with isolated nonspecific populations of bladder innervating DRG, we see nonspecific sensitization by supernatants from infected bladders (2). Clinically, studies in patients confirm that specific targeting of sensory nerves via the bladder lumen with phenazopyridine, lidocaine, and RTX preferentially and effectively relieve hypersensitivity symptoms including urgency and bladder pain without major impacts on normal bladder function (44–49), further supporting a major and specific role for mucosal afferents in bladder pain signaling.

Irritative sensations driven by the activation of peripheral sensory nerves are crucial signifiers of infection and inflammation that are necessary for directing behavioral responses to protect against threats across various organs including the lungs and gastrointestinal tract (50, 51). Urinary frequency has been proposed to act as an analogous defensive behavioral response that aids the innate immune response in bacterial clearance through rapid expulsion of bacteria that would otherwise be stored and growing in the bladder reservoir (18, 19). Clinically, disruptions to bladder sensory or motor functions such as those observed in persons with neurogenic lower urinary tract dysfunction caused by Parkinson’s disease, multiple sclerosis, spinal cord injury, and diabetic neuropathy are associated with increased susceptibility to both initial and recurrent UTI’s, and an increased risk developing severe UTI’s (52–56). In this study, we show that mucosal afferents are essential for driving the development of urinary frequency during UTI in mice. Furthermore, in the absence of UTI-induced urinary frequency following mucosal denervation, we observed greater bacterial burden in the bladder wall across two UPEC clinical isolates, providing evidence of a mechanistic link between altered bladder sensation and UTI burden. However, whether this represents a generalized physiological mechanism during UTI requires further exploration, including assessment of avirulent *E. coli* strains, UPEC with attenuated virulence genes, and non-UPEC species, such as with *Klebsiella*, *Staphylococcus*, and *Candida*.

In addition to disrupting the development of increased voiding as a protective response to UTI, mucosal denervation with RTX may also impact infection severity and dissemination to the kidneys through a range of nonsensory and voiding-independent mechanisms mediated by altered local release of neuropeptides such as CGRP and Substance P (57). Neuropeptides can act directly on immune and epithelial cells to modulate host defense, including the regulation of antimicrobial peptide production, intracellular signaling pathways, epithelial cell exfoliation, and immune cell recruitment and function (31, 57–60). Importantly, depending on the context these immunoregulatory effects can be either protective or detrimental, enhancing pathogen clearance or impairing host defense (31, 57, 59, 61–68). In this study, RTX resulted in an almost complete loss of mucosal CGRP, accompanied by an increased relative abundance of neutrophils in the absence of changes in other immune cell populations. Neuronal CGRP can act directly on immune cells to modulate cytokine release and neutrophil migration (31, 57, 59) raising the possibility that its depletion may have directly influenced neutrophil recruitment in our model. However, as neutrophils are the primary effector cells responsible for bacterial clearance and their recruitment typically scales with infection severity (69), the observed increase is more consistent with a response to the elevated bacterial burden that we observed than with enhanced host defense (69, 70).

Urothelial exfoliation in response to UPEC invasion represents another critical, voiding-independent host-defense mechanism that reduces bacterial burden and limits dissemination (71). Although we did not directly assess the impact of mucosal denervation on exfoliation, previous studies demonstrate that systemic loss of TRPV1^+^ neurons and local CGRP signaling can alter bacterial invasion dynamics (72). These studies raise the possibility that changes in urothelial function may contribute to the increased bacterial burden and dissemination observed in our study following mucosal denervation. More recently, however, CGRP produced by group 2 innate lymphoid cells, rather than neurons, has been shown to be the crucial driver of urothelial exfoliation during the early phase of UTI (60). Nevertheless, loss of neuronal CGRP may still influence exfoliation indirectly, for example through modulation of immune–urothelial cross-talk. Whether reduced neuronal CGRP in our model alters urothelial exfoliation dynamics, and whether this occurs via direct urothelial effects or indirectly through altered immune cell function, remains unknown. Future studies are required to disentangle neuronal versus immune-derived neuropeptide contributions to urothelial antimicrobial signaling or exfoliation dynamics during UTI without the confounding effects of global mucosal denervation.

## Conclusions

Together, our data implicate bladder mucosal afferents as key sensors of pathogenic challenge and reveal a previously unrecognized mechanism that drives behavioral responses to reduce the burden and extent of UTI.

## Materials and Methods

Complete methods are available in *SI Appendix, Materials and Methods*.

### Animals.

Female mice aged 6 to 8 wk of age were used for all experiments and were randomly assigned to experimental procedures and were single housed where necessary for experimental design.

Na_v_1.8^-Cre-TdTomato^ mice were created as previously described (73) by crossing Na_v_1.8^-Cre^ mice with TdTomato reporter floxed mice. Transgenic mice on a C57Bl/6 background expressing Cre recombinase under control of the Na_V_1.8 promoter were generously donated by A/Prof Wendy Imlach (Monash University, Melbourne, Australia) and were originally generated from JAX stock #036564 (74). Na_V_1.8-Cre mice were crossed with tdTomato reporter mice gifted by Susan Woods (SAHMRI, Adelaide, Australia); these mice possess a loxP-flanked Stop cassette that is excised in Cre-expressing neurons, allowing tdTomato production in cells expressing Na_V_1.8-Cre only. As it has been previously shown that Na_v_1.8 is present in the vast majority of bladder neurons (75), this mouse provides an effective and specific sensory neuron marker in the bladder. Furthermore, a Nav1.8-tdTomato reporter system combined with CGRP staining has become a standard approach in recent seminal studies of sensory nerve ablation with RTX to overcome limitations in immunostaining for TRPV1 (63, 68, 76).

### Resiniferatoxin Treatments.

6 to 8 wk old female C57BL/6J or Na_v_1.8^-Cre-TdTomato^ mice were anaesthetized under isoflurane (3%/1 L O_2_) and 100 µL of 30 µM RTX was infused into the bladder for 30 min. Mice received the same infusions across three consecutive days and were utilized for downstream experiments 7 d after the final infusion.

### Quantification of Bladder Nerve Density.

Bladders were surgically removed from RTX and control mice and fixed in 4% PFA (Sigma-Aldrich, #158127) for 18 to 20 h at 4 °C before cryoprotection in 30% sucrose/phosphate buffer for 18 to 20 h at 4 °C and to freezing in 100% Optimum Cutting Temperature (OCT) Compound (Sakura Finetek) using liquid nitrogen cooled 2-methylbutane (Sigma-Aldrich, M32631). Bladders were cryosectioned at 40 µm thickness and placed on gelatine-coated slides for immunofluorescence labeling with primary CGRP antibody (1:400) (Sigma-Aldrich, cat #PC205L). Sections were washed three times with PBSTx_0.2%_ for 5 min each prior to incubation for 1 h with chicken anti-rabbit-AF488 secondary antibody (1:200) (Thermo Fisher Scientific-Invitrogen Cat #A-21441) at room temperature for imaging using an Zeiss Axio Scan.Z1 slide scanner. Images were analyzed in ImageJ using the area tool. The integrated density (IntDen), area and %area was measured for the mucosal and detrusor layer for each mouse and were based on the TdTomato and AF488 fluorescence that was above a set threshold. The IntDen is the product of the area and mean gray value, the area is the area of the selection in mm^2^ and %area is the percentage of pixels in the image or selection that is above a set threshold. The IntDen, area, and %area measurements were averaged across each bladder region (opening, middle, and dome). Consistency of area taken for analysis across groups was confirmed by assessing perimeter of mucosa and detrusor using ImageJ (*SI Appendix*, Fig. S13).

### CGRP ELISA.

Bladders were removed from RTX and SHAM treated mice and mucosa and detrusor were rapidly separated by fine dissection and snap frozen in liquid nitrogen. The CGRP (rat) ELISA kit (Bertin Bioreagent, cat. A05482) was used to quantify the amount of CGRP within the supernatants as per the manufacturer’s instructions.

### Flow Cytometry.

Bladders were surgically removed and vertically bisected along the urethra toward the bladder dome and placed in an Eppendorf tube containing 1 mL of RPBMI1640 medium containing digest buffer (1 mg/mL collagenase + 100 µg/mL DNAse I) (Thermo Fisher Scientific™–Gibco™, cat. 17100017; Bio-Rad, cat. 7326828) to generate single cell suspensions. Cells were incubated in 50 µL of a previously prepared primary antibody cocktail (Table 1) and left to incubate for 30 min on ice in the dark. Cells were resuspended in 50 µL of secondary antibody (Streptavidin-PE-CSF594; BD Biosciences), washed twice with FACs buffer and then resuspended in preparation for flow cytometry analysis. Flow cytometry was performed using the BD LSRFortessaTM X-20 (BD Bioscience) at the SAHMRI flow cytometry core facility using the gating strategy shown in *SI Appendix*, Fig. S14. Subsequent data analysis was conducted using FlowJo software version 10.8.1.

**Table 1.** Pan immune cell bladder antibody panel

Target	Fluorophore	Company	Cat#	Dilution	Marker/function
Fc Block	–	BD Biosciences	553141	1:400	Used to block nonspecific binding of antibodies to cells
CD45.2	APC-Cy7	BD Biosciences	560694	1:400	Expressed on many different types of immune cells
CD19	PerCP-Cy5.5	BD Biosciences	551001	1:200	Specific to B cells
CD3e	PerCP-Cy5.5	BD Biosciences	551163	1:200	Present on T cells
CD4	BV510	BD Biosciences	563106	1:200	Found on CD4 T cells, a subtype of helper T cells
CD8a	BUV395	BD Biosciences	563786	1:200	Found on CD8 T cells, a subtype of cytotoxic T cells
NK-1.1	APC	BD Biosciences	550627	1:200	A specific marker for Natural Killer (NK) cells
Ly-6G	Pe-Cy7	BD Biosciences	560601	1:400	Specific marker for neutrophils
CD11b	PE	BD Biosciences	557397	1:800	Specific to myeloid cells
CD11c	Biotin	Miltenyi	130-101-929	1:50	Expressed on monocytes, granulocytes, and tissue macrophages
MHCII (I-A/I-E)	BV711	BD Biosciences	563414	1:1,500	Found on macrophages, B cells, and other myeloid cells; indicates activation in myeloid cells
Ly-6C	FITC	BD Biosciences	553104	1:400	Specific to monocytes
F4/80	BV421	BD Biosciences	565411	1:400	Specific to macrophages

### Hematoxylin and Eosin Staining.

Bladders were collected and fixed in 4% PFA for 24 h, rinsed, and transferred to 70% ethanol. Bladders were then embedded in paraffin and serially sectioned from the middle of the bladder toward the dome in 5 µm sections and subsequently stained with H&E by the Flinders Microscopy and Microanalysis Core Facility and scored to compare inflammation between the bladder mucosa and muscle by a researcher blinded to the conditions. Sections were scored according to Table 2.

**Table 2.** Histopathological score criteria for bladder muscle and mucosa

Score	Tissue damage		Oedema		Hemorrhage
	Muscle (fibrosis)	Mucosa (sloughing)	Muscle	Mucosa	
0	Full muscle integrity	Full urothelium integrity	No edema present	No edema present	No free erythrocytes
1	Minimal fibrosis (<25% disruption of muscle fibers)	Sloughed urothelium (<25% of section)	Slight separation of muscle fibers	Slight separation in urothelial cells	<10 erythrocytes
2	Moderate fibrosis (25 to 50% disruption)	Sloughed urothelium (25 to 50% of section)	Moderate separation of muscle fibers	Moderate separation, expanded submucosa	10 to 30 erythrocytes
3	Severe fibrosis (>50% of muscle fiber disruption)	Sloughed urothelium (>50% of section)	Severe stretching/distortion of muscle fibers	Severe separation, stretching/distortion of urothelial cells	>30 erythrocytes

### Ex Vivo Bladder Pelvic Afferent Nerve Recordings.

#### Flat-sheet bladder preparation.

To individually evaluate the direct activity of the bladder mucosal and muscular nerves, ex vivo flat-sheet bladder afferent recordings were performed (43, 77, 78). After mice were humanely killed via CO_2_ inhalation, nerve trunks entering the trigone area of the bladder between the left (or right) ureter and urethra were carefully isolated from surrounding connective tissue using a tungsten needle. The nerve trunks were pinned loosely with 50 µm gold-plated tungsten pins, and a ring of paraffin was placed around and over the nerves; this formed the foundation for making a paraffin oil bubble for electrical isolation and recording of electrical activity. The opposite edge of the bladder was attached via a hook and cantilever system, to an isotonic transducer (Harvard Bioscience 52-9511, S Natick, MA, USA) for stretching imposed by loads (1 to 5 g) and simultaneous measuring of the preparation lengthening. The preparation was rested for 30 min before commencing experiments. Electrical signals were amplified (DAM 80, WPI), filtered via band-pass filter (BPF-932, CWE, USA, band pass 10 Hz-10 kHz) and recorded by a computer at 20 kHz with a Micro 1401-4 data acquisition system (CED, UK). Single units were discriminated offline by using Spike 2 version 10 software (CED, UK).

#### Characterization of bladder afferent subtypes.

The current study focused on the three major functional classes of bladder afferents: mucosal, muscular-mucosal, and muscular afferents. Afferent sensitivity to stroking was determined using calibrated von Frey hairs (10, 50, and 100 mg) stroked across the receptive field at a rate of 5 mm/s five times. The middle three strokes were used for analysis. Afferent sensitivity to bladder stretch was determined by imposed loads (1, 3, and 5 g) applied via the cantilever system for 10 s with a 1 min interval between each load. Afferent subtypes were differentiated based on their well characterized responsiveness to stroking and stretching (12, 14). Mucosal afferents respond to light mucosal stroking with von Frey Hairs but not to bladder stretch imposed by a load. Muscular afferents do not respond to von Frey hair stroking but do respond to bladder stretch. Muscular-mucosal afferents respond to both mucosal stroking by von Frey hairs and to bladder stretch.

### Whole Bladder Pelvic Afferent Recordings.

To evaluate the nerve activity of the bladder during distension, ex vivo whole bladder afferent recordings were used as previously described (23, 29, 73, 75, 79–81). Mice were humanely killed by CO_2_ inhalation, and the entire lower abdomen was removed and submerged in a modified organ bath under continual perfusion with gassed (95% O_2_ and 5% CO_2_) Krebs buffer at 35 °C. The bladder was catheterized (PE 50 tubing) through the urethra and connected to a syringe pump (NE-1000) to allow a controlled fill rate of 100 µL/min with saline (NaCl, 0.9%). A second catheter was inserted through the dome of the bladder, secured with silk, and connected to a pressure transducer (Digitimer; cat. NL108T2) to enable intravesical pressure recording during graded distension. Pelvic nerves, isolated from all other nerve fibers between the pelvic ganglia and the spinal cord, were dissected into fine multiunit branches, and a single branch was placed within a sealed glass pipette containing a microelectrode (WPI) attached to a Neurolog headstage (Digitimer; cat. NL100AK) to record nerve activity during bladder distension.

### Voiding Paper Assay.

Single housed mice were temporarily removed from their cages and their bedding was replaced with filter paper (Whatman; Grade 1) fixed to the bottom of the cage. Mice remained in their lined cages for 3 h, between 10 AM and 1 PM, with free access to food and water after which filter paper was collected, imaged using an ultraviolet transilluminator (Gel Doc XR+Gel Documentation System; Bio-Rad), and saved as .jpg files. Images were digitized into binary images using ImageJ software and the number and size of urine spots was determined by a researcher blinded to the conditions.

### Electronic von Frey Hair (EvFH) Testing.

The electronic von Frey Hair (EvFH) test was used to assess abdominal sensitivity to mechanical stimuli by measuring the withdrawal response to a stimulus (82, 83). The BIO-EVF4 (Bioseb) was used to conduct EvFH tests by a researcher blinded to the conditions. The minimum force applied (in grams) that elicited the abdominal withdrawal was noted as the withdrawal threshold. The pelvic area was stimulated three times over 5 min, and an average withdrawal force was calculated from the trials. The mice were tested on rotation, so each mouse was stimulated first before starting the second round of stimulations to reduce acclimation to the filament.

### Mouse Model of Urinary Tract Infection.

#### UPEC culture and infusions.

UPEC culture and infusions were performed as previously described (1) using UPEC strain CFT073 (ATCC), a commonly used strain in UTI animal models (72, 84, 85), and the human cystitis isolate UTI89 (86). Overnight cultures were subsequently grown to a final concentration of approximately 1 × 10^8^ CFU/mL for use in bladder instillations.

UPEC instillation was performed in C57BL/6J mice aged 7 to 8 wk old, 7 d after the final intravesical infusion of RTX (RTX-UTI), or PBS (UTI). Mice were anaesthetized with isoflurane, and a lubricated sterile catheter was inserted into the bladder via the urethra to remove urine. A second sterile catheter was then used to instill 50 µL of UPEC broth (1 × 10^8^ CFU/mL) for 10 min. Sham (control) mice received instillations of 50 µL 1× PBS for 10 min. Twenty-four hours after bladder infections, urine was collected by scruffing and capturing spontaneously voided urine into a sterile Eppendorf. Mice were then humanely killed via CO_2_ inhalation, and bladders and kidneys were removed for bacterial load assessment.

#### Quantification.

A researcher blinded to the experimental conditions placed bladders and kidneys into FastPrep® microfuge lysing matrix tubes (MP Biomedicals, cat. 115076200-CF) containing ice-cold sterile 1× PBS and a chrome steel bead to homogenize. Urine samples and homogenized tissues were either plated neat or following serial 10-fold dilution in sterile LB broth onto MacConkey No.3 agar (Oxoid™, cat. CM0115B), as required to obtain countable colonies. Bacterial burden was calculated as CFU/ml after a 24 h incubation.

### Statistical Analysis.

GraphPad Prism v10.2.3 (GraphPad Software, La Jolla, CA) was used for statistical analyses, using Student’s *t* tests when comparing two independent variables, or ANOVA when comparing three or more independent values. Normality tests were performed to determine whether a parametric or nonparametric statistical test should be used. If the data were normal, a parametric test was used and if not normal, a nonparametric test was used. The significance value was set at 0.05.

## Supplementary Material

Appendix 01 (PDF)

## Data Availability

Reagents used here will be shared upon reasonable request to the corresponding author. All other study data are provided in the article or *SI Appendix*.
