# Supplementary material for: Bladder mucosal afferents detect UTI and aid pathogen clearance
Source: Proc Natl Acad Sci U S A. 2026 Aug 3;123(32):e2604124123. doi: 10.1073/pnas.2604124123 (PMC13462357; doi:10.1073/pnas.2604124123)
Supplement: Supplementary file 1 [file pnas.2604124123.sapp.pdf]

Materials and Methods

Animals

Female mice aged 6-8 weeks of age were used for all experiments and were randomly assigned to experimental procedures and were single housed where necessary for experimental design.

$Na_v1.8^{-Cre-TdTomato}$ mice were created as previously described (74) by crossing $Na_v1.8^{-Cre}$ mice with TdTomato reporter floxed mice. Transgenic mice on a C57Bl/6 background expressing Cre recombinase under control of the $Na_V1.8$ promoter were generously donated by A/Prof Wendy Imlach (Monash University, Melbourne, Australia) and were originally generated from JAX stock #036564 (75). $Na_V1.8$-Cre mice were crossed with tdTomato reporter mice gifted by Prof Susan Woods (SAHMRI, Adelaide, Australia); these mice possess a loxP-flanked Stop cassette that is excised in Cre-expressing neurons, allowing tdTomato production in cells expressing $Na_V1.8$-Cre only. As it has been previously shown that $Na_v1.8$ is present in the vast majority of bladder neurons (76), this mouse provides an effective and specific sensory neuron marker in the bladder. Furthermore, a Nav1.8-tdTomato reporter system combined with CGRP staining has become a standard approach in recent seminal studies of sensory nerve ablation with RTX to overcome limitations in immunostaining for TRPV1 (64, 69, 77).

Resiniferatoxin treatments

Resiniferatoxin (RTX; 1mg) was dissolved in dimethyl sulfoxide (DMSO) to a stock concentration of 1mM. 50µl of frozen stock was diluted in 1.5ml of phosphate buffered saline (PBS) to give a 30µM working concentration to perform intravesical RTX infusions in mice. 6-8 week old female C57BL/6J or $Na_v1.8^{-Cre-TdTomato}$ mice were anaesthetised under isoflurane (3%/1L O_2) and a lubricated catheter was inserted into the bladder via the urethra. Urine was drained from the bladder followed by instillation of 100µl of 30µM RTX for 30 mins. Control mice received sham treatments consisting of bladder catheterisation and infusion of 100µl of 1X PBS. Mice received the same infusions across three consecutive days and utilised for downstream experiments 7 days after the final infusion.

Quantification of bladder nerve density

Bladders were surgically removed from RTX and control mice and fixed in 4% PFA (Sigma-Aldrich, #158127) for 18 to 20 hrs at 4°C. PFA was removed, and the tissue was cryoprotected in 30% sucrose/phosphate buffer for 18 to 20 hrs at 4°C prior to freezing in 100% Optimum Cutting Temperature (OCT) Compound (Sakura Finetek) using liquid nitrogen cooled 2-methylbutane (Sigma-Aldrich, M32631). Bladders were frozen in intermediate sized cryomolds (Tissue-Tek, Sakura Finetek) with the bladder dome orientated towards the top of the cryomold. Bladders were cryosectioned at 40μm thickness and placed on gelatine-coated slides for immunofluorescence labelling. Bladders were serially sectioned from the urethral opening to the dome over 4 slides.

Calcitonin Gene Related Peptide (CGRP) Immunohistochemistry

Bladder sections from $Na_V1.8^{-Cre-tdTomato}$ mice were air dried for 20 mins prior to three washes with 0.2% Triton X-100 (Sigma-Aldrich, cat. T8787) in 1X PBS ($PBSTx_{0.2\%}$) for 5 mins each. Sections were incubated in 5% normal chicken serum (Thermo Fisher Scientific-Gibco™, cat # C34777) diluted in $PBSTx_{0.2\%}$ for 30 mins at room temperature and then incubated with primary CGRP antibody (1:400) (Sigma-Aldrich, cat #PC205L) at room temperature for 12 to 18 hours in a moist, wet and humid environment. Sections were washed three times with $PBSTx_{0.2\%}$ for 5 mins each prior to incubation for 1 hr with chicken anti-rabbit-AF488 secondary antibody (1:200) (Thermo Fisher Scientific-Invitrogen Cat #A-21441) at room temperature. Following three 5 min washes with $PBSTx_{0.2\%}$, sections were cover slipped with Prolong Diamond Antifade Mountant with DAPI (Thermo Fisher Scientific-Invitrogen; cat. P36962). Slides were left to dry at room temperature for 24 hrs before visualisation. Bladder sections were visualised using the Zeiss Axio Scan.Z1 slide scanner at Adelaide Microscopy. Images were obtained with 1-4 manually set focus points per section at 20x magnification using the 475nm and 567nm LED module. To visualise Alexa Fluor 488 fluorescence, the 475nm module was tuned to 493nm excitation and 517nm emission settings. While TdTomato was visualised using the 567nm module tuned to 553nm excitation and 568nm emission settings. These settings were saved and used for all imaging. Images were automatically saved as a Carl Zeiss Image and viewed using Zen Blue. Images of sections from each region (opening, middle and dome) were exported as a TIFF file.

Analysis

Images were analysed in ImageJ using the area tool. The Integrated Density (IntDen), area and %area was measured for the mucosal and detrusor layer for each mouse and were based on the TdTomato and AF488 fluorescence that was above a set threshold. The IntDen is the product of the area and mean gray value, the area is the area of the selection in mm^2 and %area is the percentage of pixels in the image or selection that is above a set threshold. The IntDen, area and %area measurements were averaged across each bladder region (opening, middle and dome). Consistency of area taken for analysis across groups was confirmed by assessing perimeter of mucosa and detrusor using ImageJ (**Fig S13**).

CGRP ELISA

Bladders were removed from RTX and SHAM treated mice and mucosa and detrusor were rapidly separated by fine dissection and snap frozen in liquid nitrogen. Frozen tissues were weighed and 10µl 100% acetic acid/mg tissue was added before bead homogenised using the TissueLyserII (Qiagen, SN: 125090912) for 6 min at 30Hz. Samples were briefly centrifuged and stored at 4°C for 15 to 20 mins. Homogenates were ultracentrifuged (45000 rpm for 30 mins at 4°C) and the supernatant was carefully removed without disturbing the pellet and transferred to a clean Eppendorf tube. Bladder protein extracts were stored at -20°C until future use.

Bladder protein extracts in supernatants were quantified using the Invitrogen EZQ™ Protein Quantitation Kit (Thermo Fisher Scientific, cat. R33200) according to manufacturer's instructions. The CGRP (rat) ELISA kit (Bertin Bioreagent, cat. A05482) was used to quantify the amount of CGRP within the supernatants as per manufacturer's instructions. All bladder protein extracts were diluted to 20 mg/ml using the kit's EIA buffer to standardise the concentration of protein when loading the ELISA plate. 100ml of samples, standards, and controls (blank, non-specific binding wells, quality control wells) were plated as duplicates. The plate was incubated for 16 hrs at 4°C, rinsed with wash buffer three times, and blotted dry before Ellman's reagent was added to each well. The plate was then incubated in the dark at room temperature on an orbital shaker at 50 rpm before being read at 405 nm after 1 hr (Beckman DTX 880 Plate Reader (Beckman Coulter).

Flow cytometry

Bladders were surgically removed and vertically bisected along the urethra towards the bladder dome and placed in an Eppendorf tube containing 1ml of RPBMI1640 medium containing digest buffer (1mg/ml collagenase + 100µg/ml DNAse I) (Thermo Fisher Scientific™ - Gibco™, cat. 17100017; Bio-Rad, cat. 7326828). Bladders were cut into small pieces with dissection scissors and then placed into a shaking incubator for 1 hr at 37°C at 180 rpm. Digestion was stopped by adding 1ml of cold RPMI (Thermo Fisher Scientific™ - Gibco™, cat. 11875093) with 10% Fetal Bovine Serum (FBS) (Thermo Fisher Scientific™ - Gibco™, cat. 10100147). The tissue suspension was then pushed through a 70 µm cell strainer and then centrifuged at 400 x g for 5 mins to pellet the cells. The supernatant was then aspirated, and the cell pellet was resuspended in 800µl of BD Pharm Lyse red cell lysis buffer (Becton Dickinson (BD) Biosciences, cat. 555899) at room temperature for 90s before 1ml of RPMI with 10% FCS was added to prevent further cell lysis. Cells were centrifuged at 400 x g for 5min, and the cell pellet was resuspended in 200µl of FACS buffer (0.1% Bovine Serum Albumin (BSA) and 0.4% Ethylenediaminetetraacetic Acid (EDTA) in PBS). Resuspended cells were transferred to a 96-well round-bottom plate, with samples from each bladder going into one well, and centrifuged at 300 x g for 5 min. The supernatant was removed by gently tapping the plate before being resuspended in 50µl of a previously prepared primary antibody cocktail (Table 1) and left to incubate for 30 mins on ice in the dark. Cells were rinsed with 200µl of FACS buffer and centrifuged at 300 x g for 5 mins at 4°C. Wells were then resuspended in 50µl of secondary antibody (Steptavidin-PE-CSF594; BD Biosciences) diluted 1:500 in FACS buffer; the cells were left to incubate with the secondary antibody for 15 to 20 mins on ice in the dark. After which the cells were washed twice with 200µl of FACs buffer and then resuspended in 120µl of FACs buffer in preparation for flow cytometry analysis. Lastly, 10µl of DAPI (Sigma-Aldrich, cat. 124653) and 4µl of counting beads were added to each well. Flow cytometry was performed using the BD LSRFortessaTM X-20 (BD Bioscience) at the SAHMRI flow cytometry core facility using the gating strategy shown in **Fig S14**. Subsequent data analysis was conducted using FlowJo software version 10.8.1.

Haematoxylin and Eosin staining

Bladders were collected and fixed in 4% PFA for 24 hrs, rinsed, and transferred to 70% ethanol. Bladders were then embedded in paraffin and serially sectioned from

the middle of the bladder towards the dome in 5µm sections and subsequently stained with H&E by the Flinders Microscopy and Microanalysis Core Facility.

Bladder H&E-stained sections were visualised using a slide scanner (Olympus VS200, Olympus Corporation, Tokyo, Japan) at Flinders Microscopy and Microanalysis Core Facility. Images were obtained with 1-4 manually set focus points per section at 20x magnification using brightfield imaging settings. Sections were scored to compare inflammation between the bladder mucosa and muscle by a researcher blinded to the conditions after training by a pathologist (Prof Sonja Klebe). Sections were scored according to Table 2.

Ex vivo bladder pelvic afferent nerve recordings

Flat-sheet bladder preparation

To individually evaluate the direct activity of the bladder mucosal and muscular nerves, *ex vivo* flat-sheet bladder afferent recordings were performed (44, 78, 79). After mice were humanely killed via CO_2 inhalation, the bladder and associated connective tissue containing nerve bundles was dissected out and opened along the midline of the anterior wall from the urethra up to the apex into a flat sheet in Krebs buffer solution (composition in mmol/L: 118.4 NaCl, 24.9 $NaHCO_3$, 1.9 $CaCl_2$, 1.2 $MgSO_4$, 4.7 KCl, 1.2 KH_2PO_4, and 11.7 glucose) bubbled in 95% oxygen and in 5% carbon dioxide. Nerve trunks entering the trigone area of the bladder between the left (or right) ureter and urethra were carefully isolated from surrounding connective tissue using a tungsten needle. The bladder was then orientated into a rectangular shape (approximately 12mm wide by 15mm long), encompassing the bladder trigone and body and pinned mucosal side up with the nerves along one edge in a 22ml organ bath continuously perfused with warmed 34°C Krebs (3ml/min). The nerve trunks were pinned loosely with 50µm gold-plated tungsten pins, and a ring of paraffin was placed around and over the nerves; this formed the foundation for making a paraffin oil bubble for electrical isolation and recording of electrical activity. The opposite edge of the bladder was attached via a hook and cantilever system, to an isotonic transducer (Harvard Bioscience 52-9511, S Natick, MA, USA) for stretching imposed by loads (1-5g) and simultaneous measuring of the preparation lengthening. The preparation was rested for 30 min before commencing experiments. Electrical signals were amplified (DAM 80, WPI), filtered via band-pass filter (BPF-932, CWE, USA, band pass 10Hz-10kHz) and recorded by a computer at 20kHz with a Micro 1401-4 data acquisition

system (CED, UK). Single units were discriminated offline by using Spike 2 version 10 software (CED, UK).

Characterisation of bladder afferent subtypes

The current study focused on the three major functional classes of bladder afferents: mucosal, muscular-mucosal, and muscular afferents. Afferent sensitivity to stroking was determined using calibrated von Frey hairs (10, 50 and 100 mg) stroked across the receptive field at a rate of 5mm/s 5 times. The middle three strokes were used for analysis. Afferent sensitivity to bladder stretch was determined by imposed loads (1, 3 and 5g) applied via the cantilever system for 10s with a 1 min interval between each load. Afferent subtypes were differentiated based on their well characterised responsiveness to stroking and stretching (13, 15). Mucosal afferents respond to light mucosal stroking with von Frey Hairs but not to bladder stretch imposed by a load. Muscular afferents do not respond to von Frey hair stroking but do respond to bladder stretch. Muscular-mucosal afferents respond to both mucosal stroking by von Frey hairs and to bladder stretch.

Analysis of bladder afferent activity

Nerve recording activity was analysed offline using Spike2 software. Single units were first discriminated using defined parameters in Spike2. To analyse spontaneous activity or stretch the 'rate' channel mode was selected with 0.5s time width, while for stroking activity the 'lines' channel mode was selected. The total number of units within a stroking or stretching event was calculated.

Whole bladder pelvic afferent recordings

To evaluate the nerve activity of the bladder during distension, *ex vivo* whole bladder afferent recordings were used as previously described (24, 33, 74, 76, 80-82). Mice were humanely killed by CO_2 inhalation, and the entire lower abdomen was removed and submerged in a modified organ bath under continual perfusion with gassed (95% O_2 and 5% CO_2) Krebs buffer at 35°C. The bladder, urethra, and ureters were exposed by removing excess tissue. Ureters were tied with 4-0 perma-hand silk (Ethicon; cat. LA53G). The bladder was catheterised (PE 50 tubing) through the urethra and connected to a syringe pump (NE-1000) to allow a controlled fill rate of

100μl/minute with saline (NaCl, 0.9%). A second catheter was inserted through the dome of the bladder, secured with silk, and connected to a pressure transducer (Digitimer; cat. NL108T2) to enable intravesical pressure recording during graded distension. Pelvic nerves, isolated from all other nerve fibres between the pelvic ganglia and the spinal cord, were dissected into fine multiunit branches, and a single branch was placed within a sealed glass pipette containing a microelectrode (WPI) attached to a Neurolog headstage (Digitimer; cat. NL100AK). Nerve activity was amplified (NL104), filtered (NL 125/126, bandpass 50-5000 Hz, Neurolog; Digitimer), and digitised (Cambridge Electronic Design; cat. CED 1401) to a PC for offline analysis using Spike2 software. The number of action potentials crossing a preset threshold at twice the background electrical noise was determined per second to quantify the afferent response. Single-unit analysis was performed offline by matching individual spike waveforms through linear interpolation using Spike2 version 5.18 software. Afferent units were deemed 'low threshold' if continuous action potential firing was elicited at less than 16mmHg. By contrast, 'high-threshold' afferents displayed continuous action potential firing only when pressures exceed 16mmHg.

Afferent recording experimental protocols

At the start of each afferent recording experiment, control bladder distensions were performed with intravesical infusion of saline at a rate of 100μl/min to a maximum pressure of 50 mmHg at 10 min intervals to assess the viability of the preparation and reproducibility of the intravesical pressure and neuronal responses to distension. The volume in the bladder was extrapolated from the known fill rate (100μl/min) and the time taken (s) to reach maximum pressure (50 mmHg). Compliance was determined by plotting intravesical pressure against the calculated volume.

Analysis of bladder afferent activity

Nerve recording activity was analysed offline using Spike2 software. Afferent activity from multiunit nerve recordings was determined using established scripts as the 'rate' of action potential firing above the baseline threshold, which was set at twice the background electrical noise. Single units were first discriminated using defined parameters in Spike2 that were consistent across all experiments. Afferent activity from single units was assessed as 'rate' of firing per unit pressure

Voiding paper assay

Voiding paper assay is a micturition assessment tool that provides information about changes in urinary behaviour and was performed as previously described (83-85). Single housed mice were temporarily removed from their cages and their bedding was replaced with filter paper (Whatman; Grade 1) fixed to the bottom of the cage. Mice remained in their lined cages for 3 hrs, between 10AM and 1PM, with free access to food and water after which filter paper was collected, imaged using an ultraviolet transilluminator (Gel Doc XR+ Gel Documentation System; Bio-Rad), and saved as .jpg files. Images were digitised into binary images using ImageJ software and the number and size of urine spots was determined by a researcher blinded to the conditions using pre-set thresholds within the ImageJ software to identify all urine spots and exclude background. For the purposes of this study, the total number of urine spots per filter paper were quantified and the number of small (500 to 100,000 pixels) and large (>100000 pixels) spots were stratified.

Electronic von Frey Hair (EvFH) testing

The electronic von Frey Hair (EvFH) test was used to assess abdominal sensitivity to mechanical stimuli by measuring the withdrawal response to a stimulus (83, 84). The BIO-EVF4 (Bioseb) was used to conduct EvFH tests. Prior to beginning EvFH tests, mice were habituated to the EvFH enclosure and placed on an elevated wire rack, for 30 mins each day, across 2 days by a researcher blinded to the conditions. On the day of testing, the same researcher moved mice to the testing room and allowed them acclimatise for a minimum of 15 mins. Animals were individually placed on the wire rack within the enclosures and left undisturbed for a minimum of 15 mins. The EvFH unit was fitted with a semi-flexible tip and zeroed. The force threshold, the minimum force required for the instrument to commence recording, was established as 0.70g within the BIO-CIS software. Once the animal was still and quiet, the force transducer was applied perpendicularly to the animal's lower abdomen from below. Force was gradually and linearly increased until a clear withdrawal response was recorded. The minimum force applied (in grams) that elicited the abdominal withdrawal was noted as the withdrawal threshold. The pelvic area was stimulated three times over 5 minutes, and an average withdrawal force was calculated from the trials. The mice were tested on rotation, so each mouse was stimulated first before starting the second round of stimulations to reduce acclimation to the filament.

Mouse model of Urinary Tract Infection

UPEC culture and infusions

UPEC culture and infusions were performed as previously described (1). Briefly, frozen stocks of UPEC strain CFT073 (ATCC), a commonly used strain in UTI animal models (73, 85, 86), and the human cystitis isolate UTI89 (87) were streaked onto Luria-Bertani (LB) agar and incubated overnight at 37 °C. Single colonies were then inoculated into sterile LB broth and grown overnight at 37 °C in an orbital shaking incubator at 200 rpm. Overnight cultures were subsequently grown to a final concentration of approximately 1 x 10^8 CFU/ml for use in instillations.

UPEC instillation was performed in C57BL/6J mice aged 7-8 weeks old, 7 days after the final intravesical infusion of RTX (RTX-UTI), or PBS (UTI). Mice were anaesthetised with isoflurane, and a lubricated sterile catheter was inserted into the bladder via the urethra to remove urine. A second sterile catheter was then used to instill 50 µl of UPEC broth (1 x 10^8 CFU/ml) for 10 min. After 10 min, the catheter was gently removed from the urethra, leaving the inoculum in the bladder. Sham (control) mice received instillations of 50 µl 1X PBS for 10 min. Twenty-four hours after bladder infections, urine was collected by scruffing and capturing spontaneously voided urine into a sterile Eppendorf. Mice were then humanely killed via CO_2 inhalation, and bladders and kidneys were removed for bacterial load assessment.

Quantification

A researcher blinded to the experimental conditions placed bladders and kidneys into FastPrep® microfuge lysing matrix tubes (MP Biomedicals, cat. 115076200-CF) containing ice-cold sterile 1X PBS and a chrome steel bead. Tissues were homogenised using the FastPrep-24™ (MP Biomedicals) for 40-80 s at 4.0 m/s before plating. Urine samples and homogenised tissues were either plated neat or following serial 10-fold dilution in sterile LB broth, as required to obtain countable colonies. From each selected dilution, 100 µl was plated in duplicate onto MacConkey No.3 agar (Oxoid™, cat. CM0115B) and incubated at 37 °C for 24 hr. Bacterial burden was calculated as CFU/ml.

Figure S1

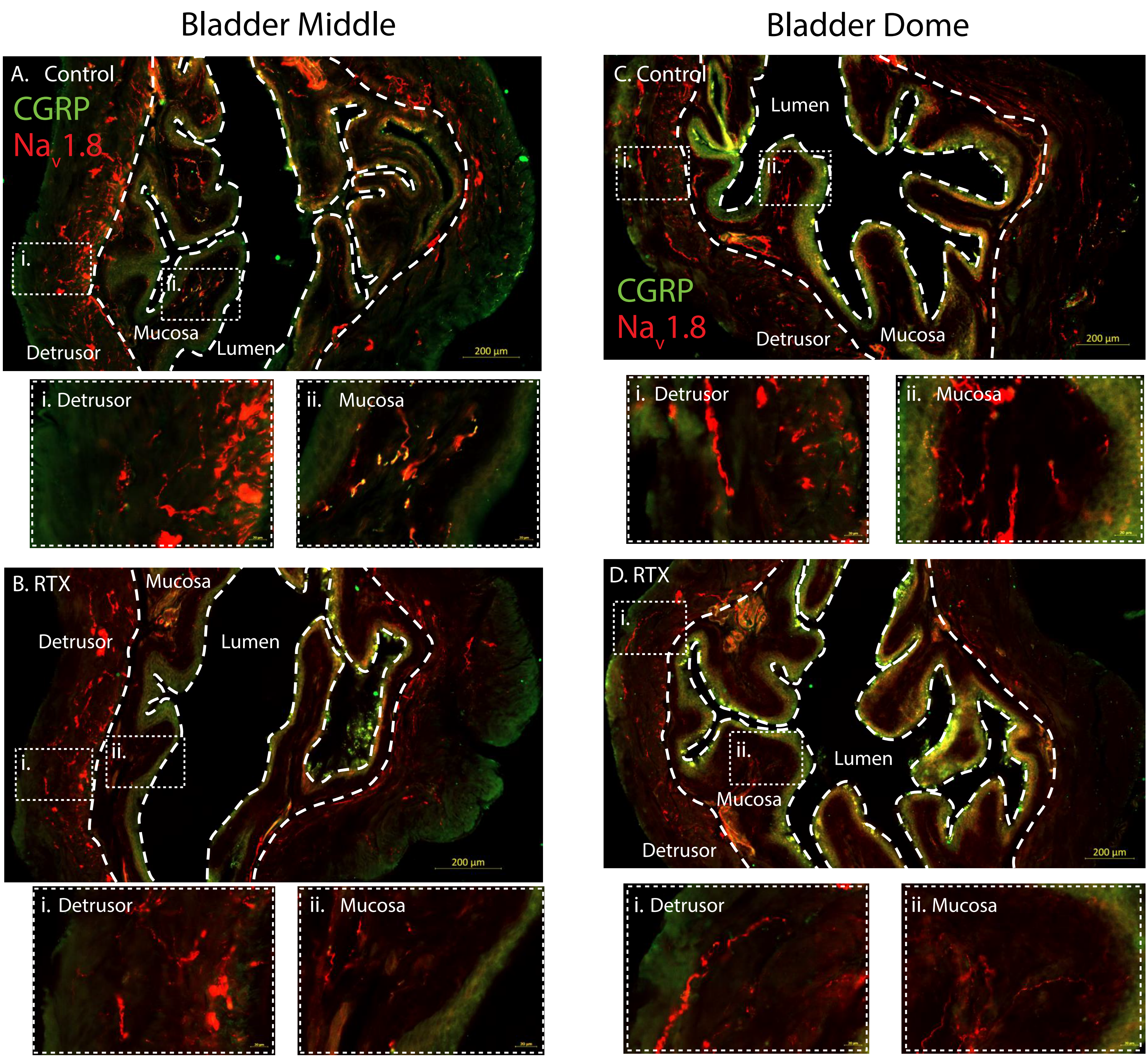

Figure S1; Labelling of $Na_v1.8^{-Cre\text{-}tdTomato}$ bladder with calcitonin gene-related peptide (CGRP) from control- and resiniferatoxin (RTX)-treated mice. Representative photomicrographs showing the distribution of CGRP-immunoreactive fibres (green) and Na_v1.8 expressing fibres (red) in cross-sections of the bladder from the middle (**A, B**) and dome (**C, D**) regions of control (**A, C**) and RTX-treated (**B, D**) mice. Fibres were located within the detrusor and the mucosa. Closeups of the afferent fibres within the detrusor (i) and mucosa (ii) are indicated by dashed boxes. Scale bar = 200µm (overall) and 20µm (inserts).

Figure S2

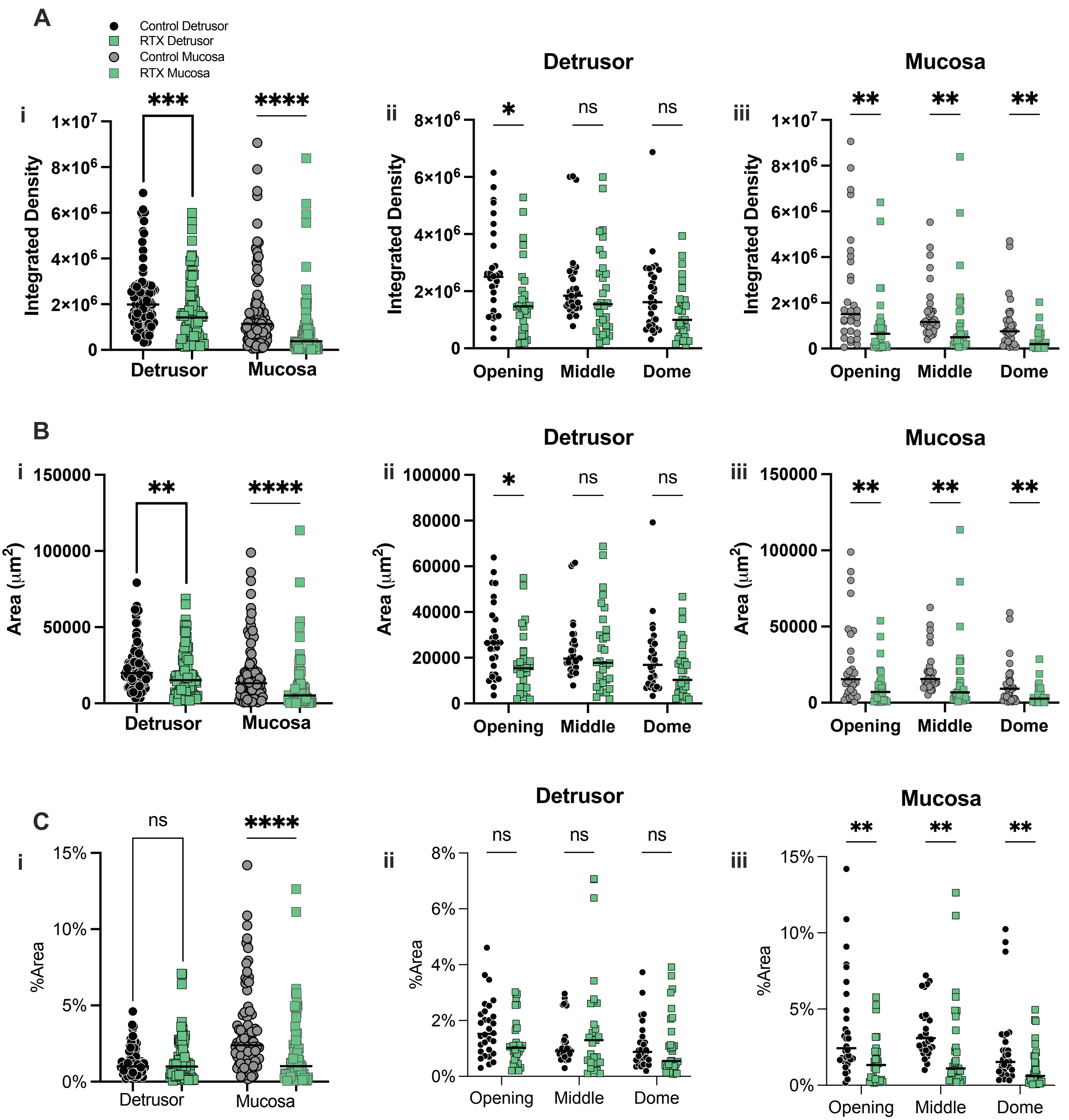

Figure S2; Integrated density, area and %area measurements of calcitonin gene-related peptide (CGRP) fluorescence within control and resiniferatoxin (RTX)-treated bladders.
Quantitative data from cross-sections of bladders from control and RTX-treated $Na_v1.8^{-Cre-tdTomato}$ mice immunostained with CGRP (represented in Fig 1, Fig S1) comparing the (A) integrated density, (B) area and (C) %area between control and RTX treated mice (green) in the detrusor (black data points) and mucosa (grey data points) across the (i) entire bladder, and at the bladder opening, middle and dome of the (ii) detrusor and (iii) mucosa layer. Individual data points represent sections (n = 90) from N = 5 mice/group. Data is presented as the median of individual values and analysed using non-parametric Mann-Whitney test, where *P < 0.05, **P < 0.01, ***P < 0.001 and ****P < 0.0001.

Figure S3

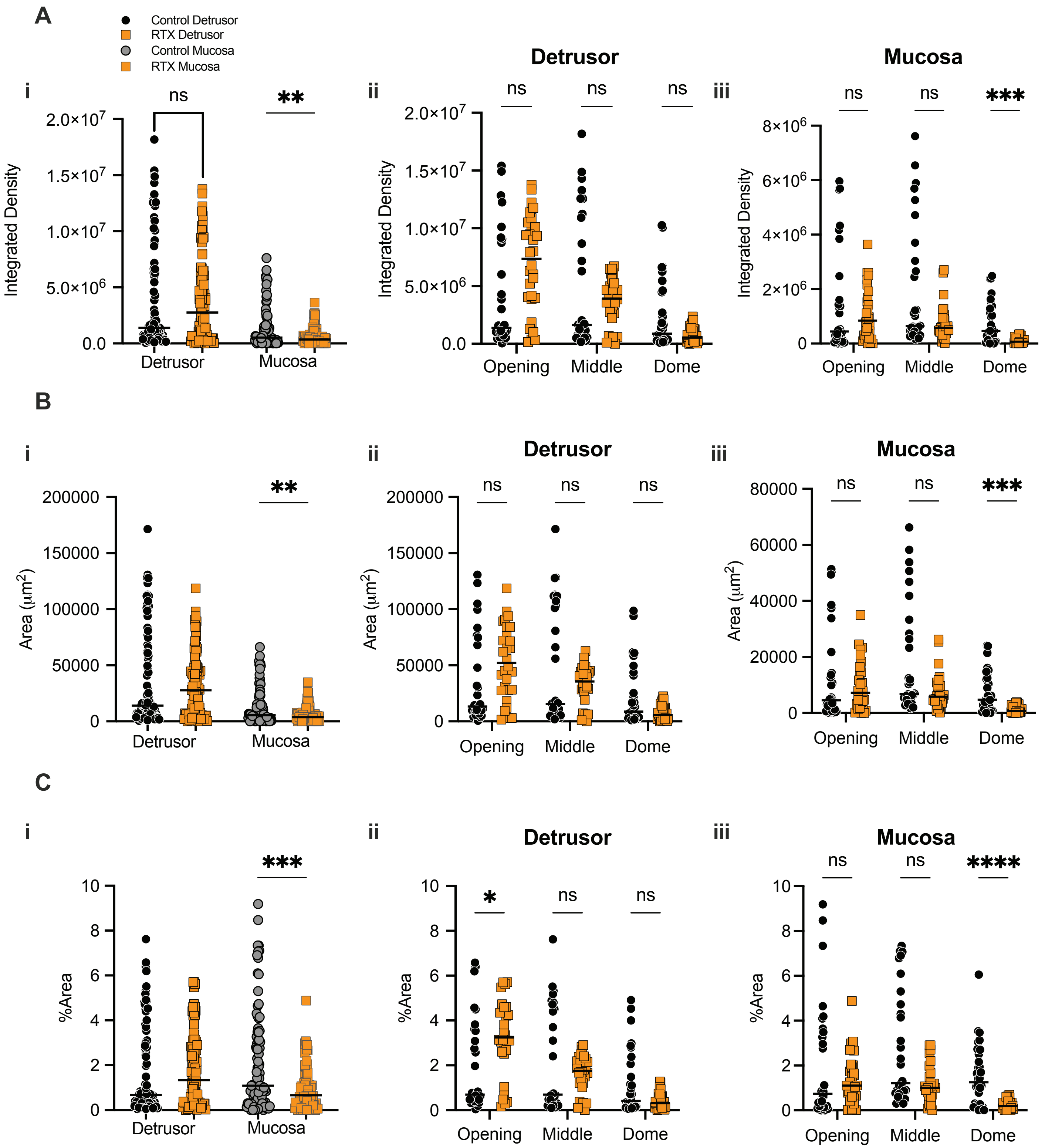

Figure S3: Integrated density, area and %area measurements of $Na_v1.8$-tdTomato fluorescence within control and resiniferatoxin (RTX)-treated bladders

Quantitative data from cross-sections of bladders from control and RTX-treated $Na_v1.8^{-Cre-tdTomato}$ mice (represented in Fig 1, Fig S1) comparing the (A) integrated density, (B) area and (C) %area between control and RTX treated mice (orange) in the detrusor (black data points) and mucosa (grey data points) across the (i) entire bladder, and at the bladder opening, middle and dome of the (ii) detrusor and (iii) mucosa layer. Individual data points represent the same sections (n = 90) from N = 5 mice/group as in Fig 1, Fig S2. Data is presented as the median of individual values and analysed using non-parametric Mann-Whitney test, where *P < 0.05, **P < 0.01, ***P < 0.001 and ****P < 0.0001.

Figure S4

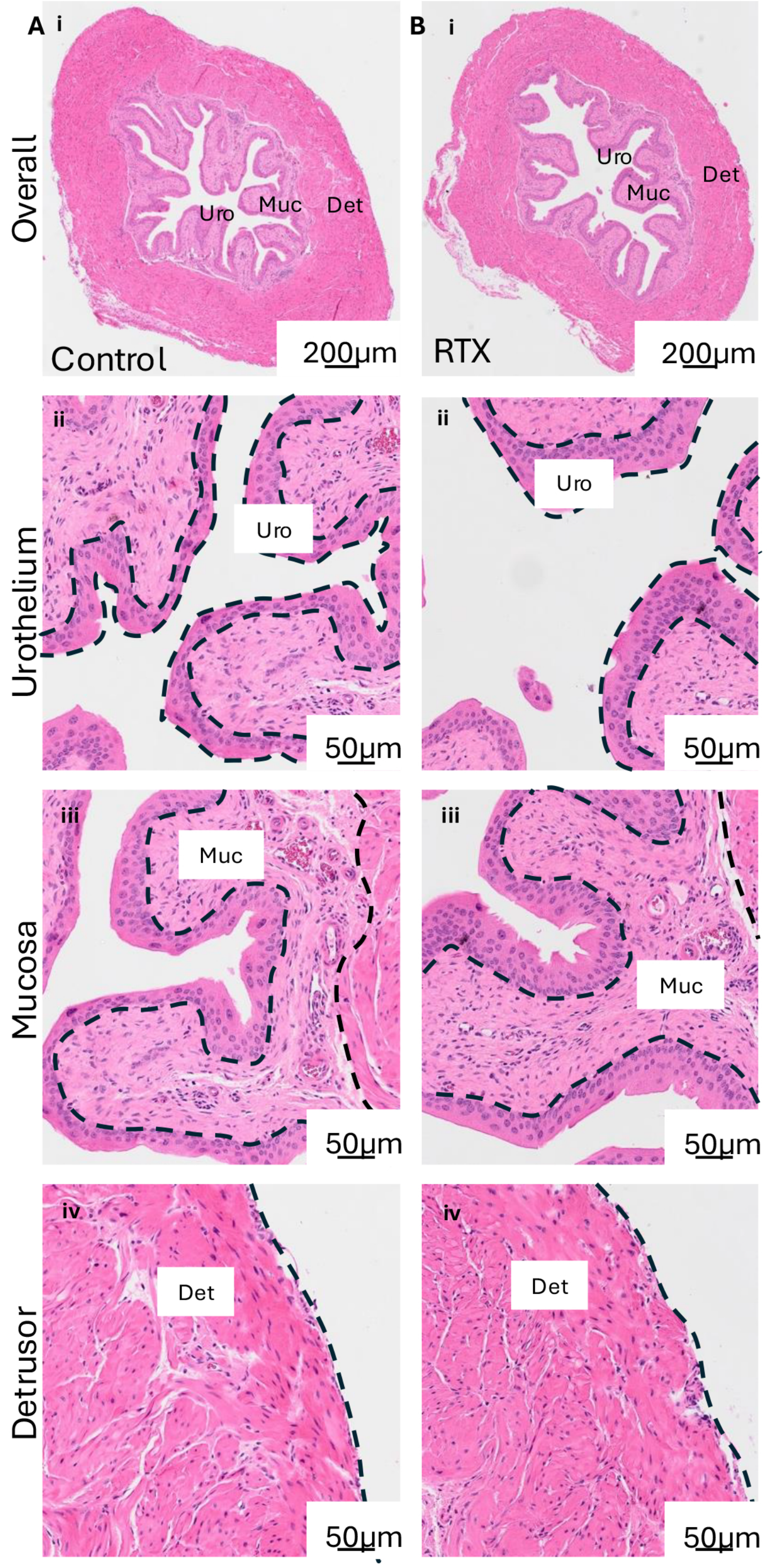

Figure S4. Representative images of hematoxylin and eosin-stained bladder sections from control and RTX-treated mice

Bladders from (A) control and (B) RTX-treated mice were fixed in 4% PFA and paraffin-sectioned at 5μ m. Sections underwent hematoxylin and eosin staining to bladder tissue integrity. Closeups of the (i) urothelium, (ii) mucosa and (iii) detrusor of the control and RTX bladders. No obvious difference in morphology was observed between control and RTX-treated bladders, including intact urothelium.

Figure S5

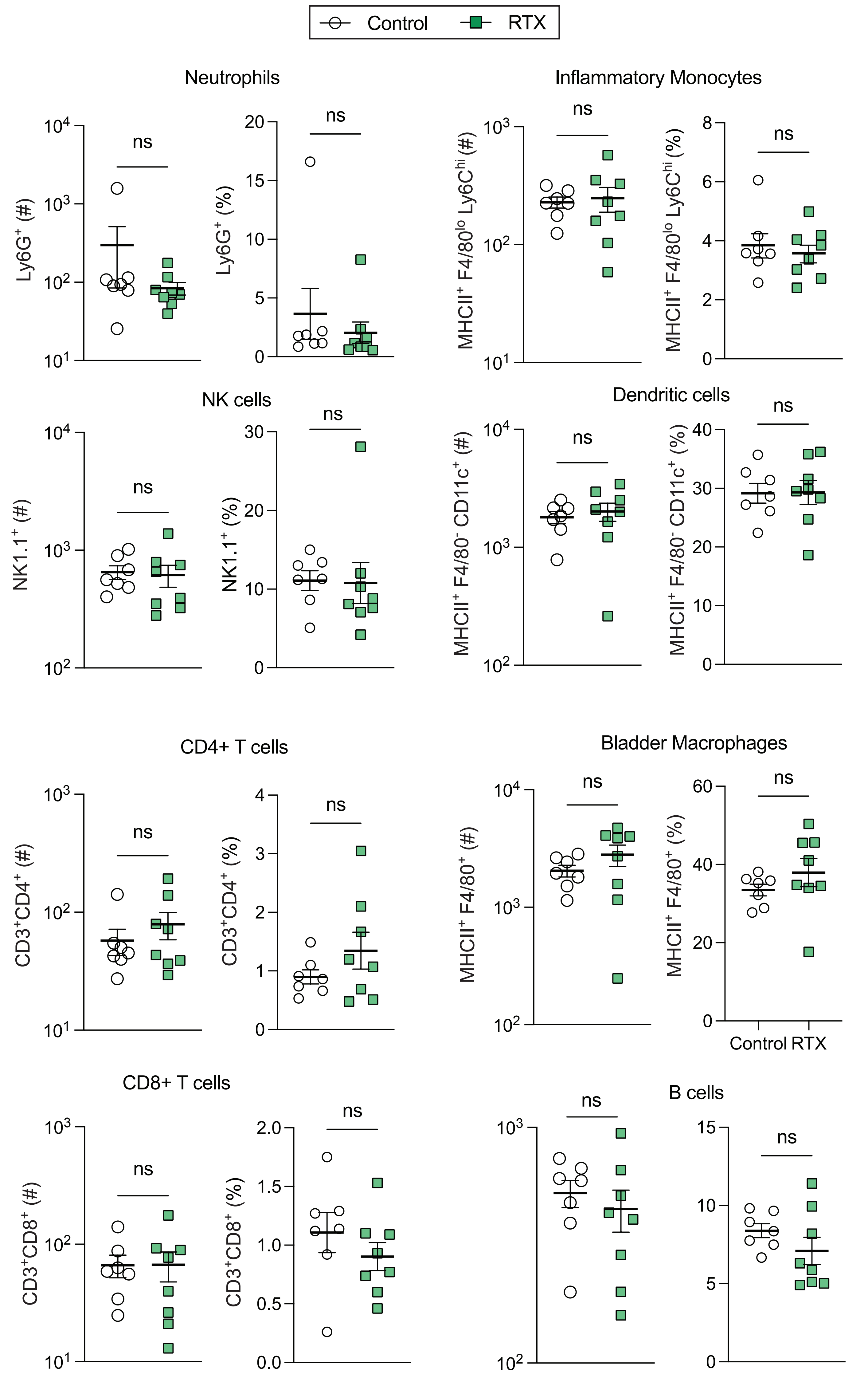

Figure S5: Quantification of immune cells from control and resiniferatoxin (RTX)-treated bladders

Bladder immune cells were sorted and quantified using flow cytometry and the gating strategy outlined in Fig S12. Neutrophils ($Ly6G^{+}$), Inflammatory monocytes ($MHCII^{+}$ $F4/80^{lo}$ $Ly6C^{hi}$), NK cells ($NK1.1^{+}$), Dendritic cells ($MHCII^{+}$ $F4/80^{-}$ $CD11c^{+}$), $CD4^{+}$ T cells ($CD3^{+}CD4^{+}$), bladder macrophages ($MHCII^{+}$ $F4/80^{+}$), $CD8^{+}$ T cells ($CD3^{+}CD8^{+}$), and B cell ($CD19^{+}$) populations seven days after RTX treatment (green data points) compared to control (black data points) bladders. Individual points represent cell counts from an individual mouse, N = 8 mice. Data was presented as mean ± SEM. Data were compared using Mann-Whitney test for neutrophils and $CD4^{+}$ T cells datasets, and Welch's t-test for bladder macrophages, inflammatory monocytes, B cells, dendritic cells, NK cells and $CD8^{+}$ T cells datasets.

Figure S6

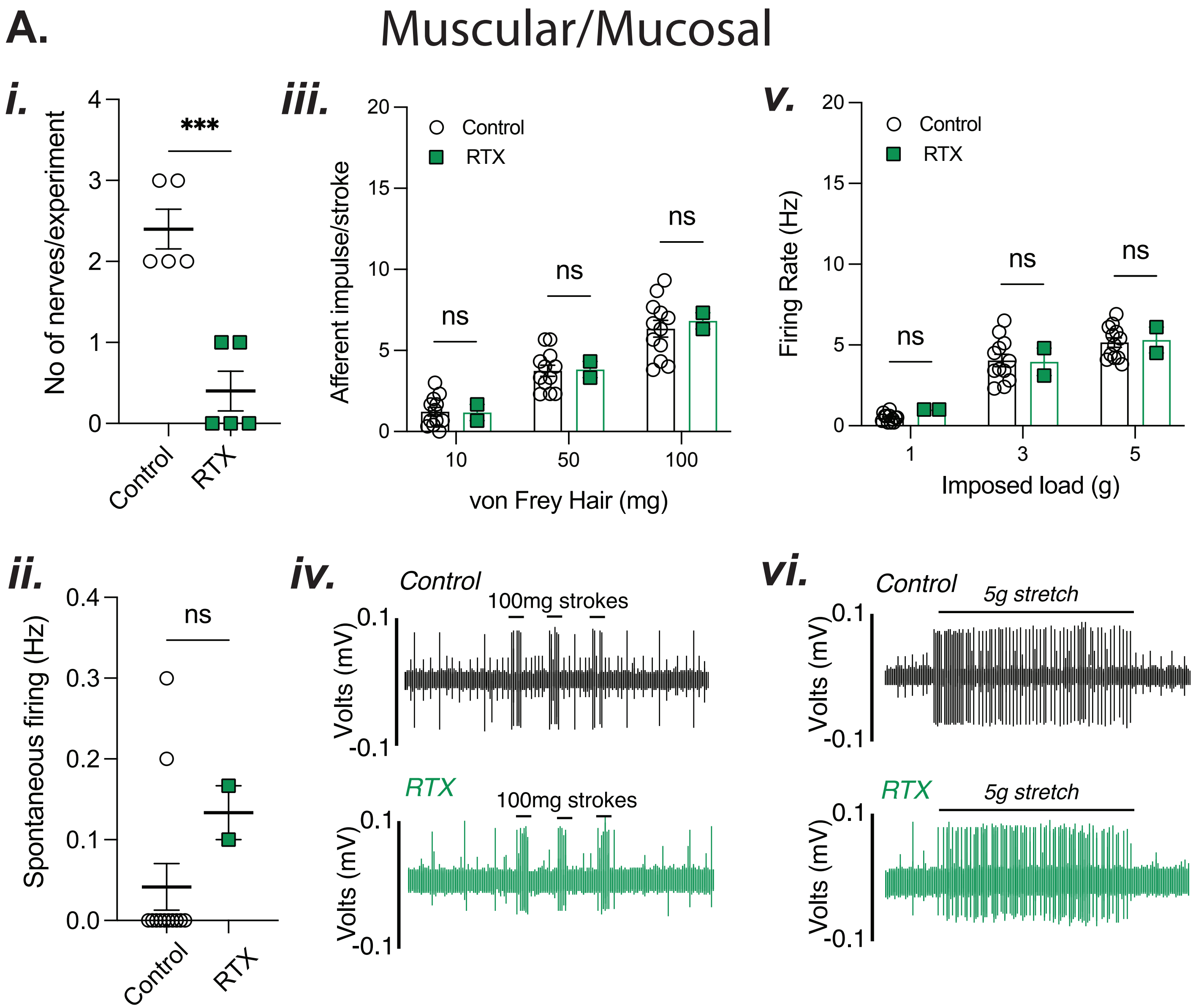

Figure S6: Muscular-mucosal afferent mechanosensitive responses from control and RTX-treated bladders

(Ai) The number of muscular/mucosal afferents identified from N=5 independent experiments/group. (Aii) Spontaneous firing frequency in remaining muscular-mucosal afferents (n=20-3 responsive units from N=5 mice/group). (Aiii) Muscular-Mucosal afferent responses to von Frey Hair stroking (10, 50, 100 mg), and (Aiv) representative response profiles of control (black) and RTX (green) treated bladders to 100 mg mucosal stroking. (Av) Muscular-Mucosal afferent responses to bladder stretch by imposed load (1, 3, 5 g), and representative response profiles of control (black) and RTX (green) treated bladders to 5g bladder stretch (Avi). Data is presented as mean ± SEM. ^{ns} P > 0.05, * p < 0.05, **p < 0.01, ***p < 0.001, and ****p < 0.0001. Statistical differences for Ai, Aii, were determined by unpaired Mann-Whitney test. For Aiii, Av, statistical differences were determined by two-way ANOVA with Geisser-Grenhouse correction and Šídák multiple comparisons.

Figure S7

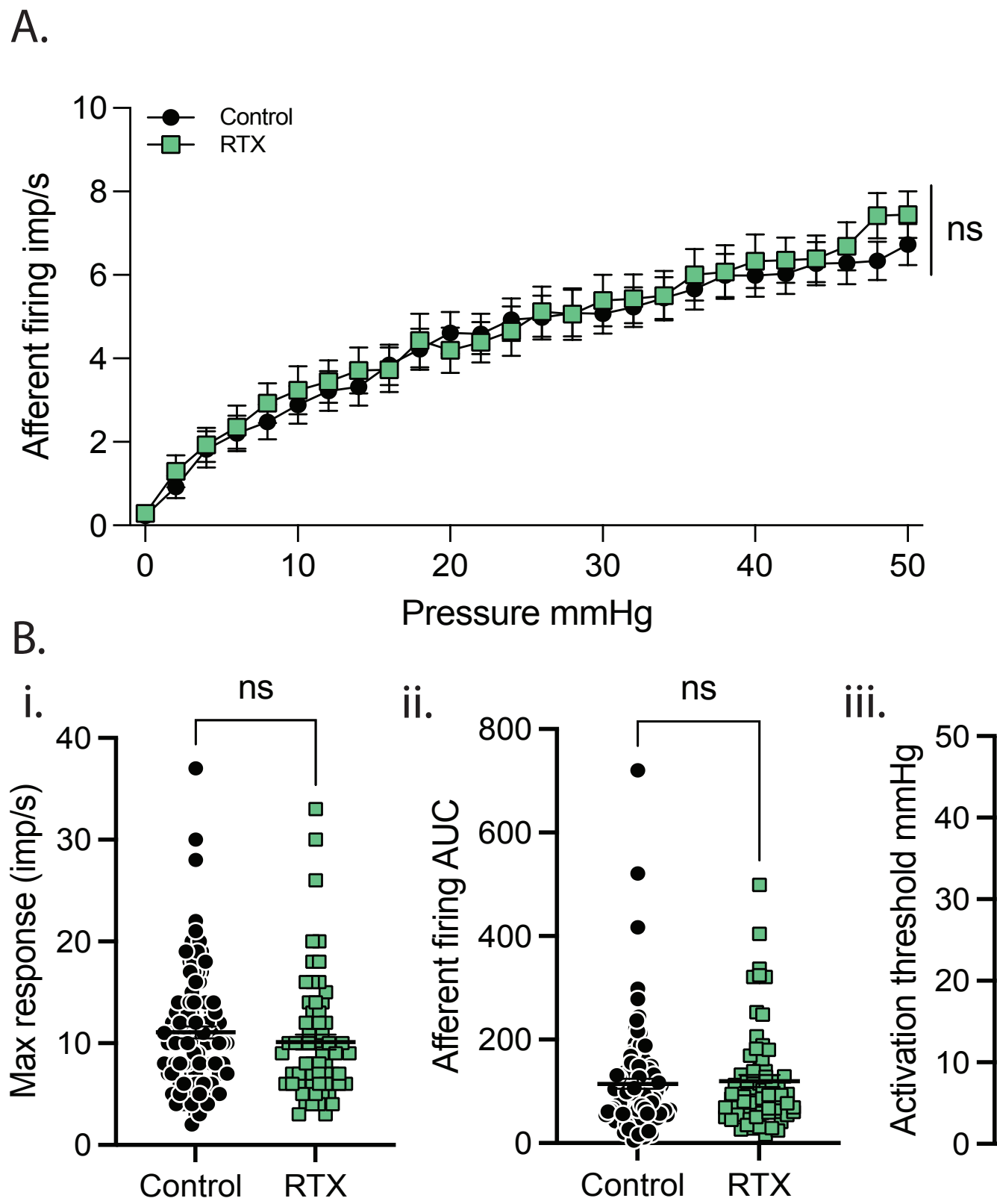

Figure S7. Resiniferatoxin (RTX) treatment does not affect single-unit bladder afferent mechanosensitive responses to distension

(A) Comparative data showing single-unit sensitivity to graded distension (0-50 mmHg) between control (black) and RTX (green) treated bladders from Fig 3 (n=7-21 units per experiment from N=5 mice/group). (B) (i) Max responses, (ii) total area under the curve (AUC) of the afferent response to distension and (iii) activation thresholds (mmHg) of individual bladder afferent units were not changed by RTX pretreatment compared to control. Data are represented as mean ± SEM, individual data points in (Bi-iii) represent values collected from each single unit. Data were compared using two-way ANOVA with Šídák multiple comparisons for A, and unpaired Mann-Whitney test for B.

Figure S8

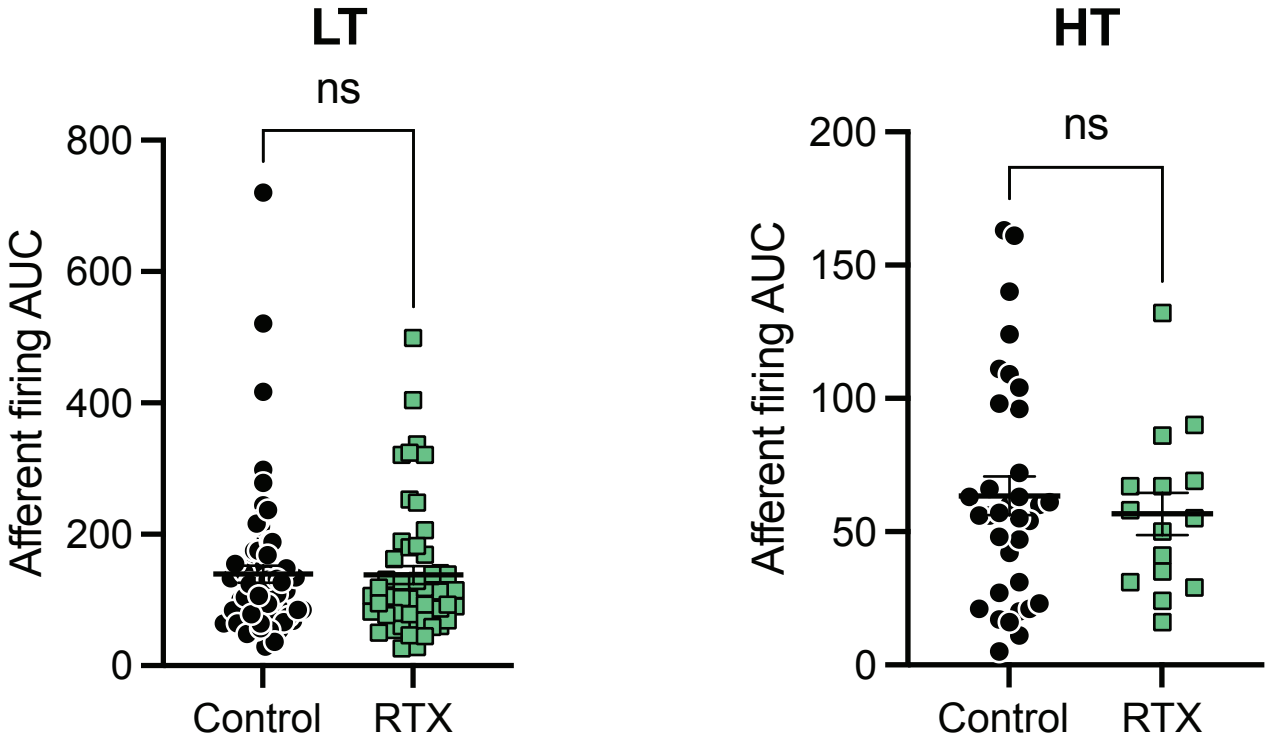

Figure S8. Resiniferatoxin (RTX) does not affect low- or high-threshold mechanosensitive bladder afferent area under the curve.

Total area under the curve (AUC) of the afferent response to distension (0-50mmHg) of individual low- (LT) and high- (HT) threshold bladder afferent units in control and RTX treated mice from Fig 3. Data are represented as mean ± SEM, individual data points represent values collected from each single unit. Data were compared using unpaired Mann-Whitney test.

Figure S9

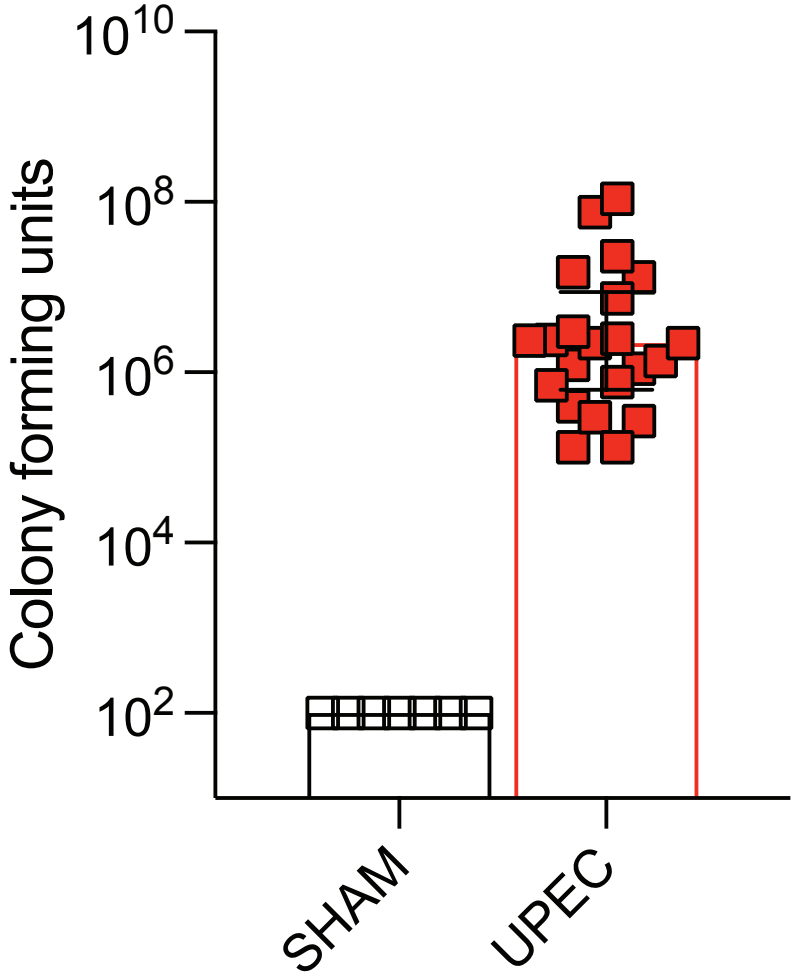

Figure S9. Urine CFU 24hrs after UPEC or Sham treatment.

UPEC CFU in urine 24hrs after transurethral bladder inoculation with either PBS (Control N=6) or UPEC strain CFT073 (N=21). Bacteria were found in the urine of UPEC but not sham treated mice.

Figure S10

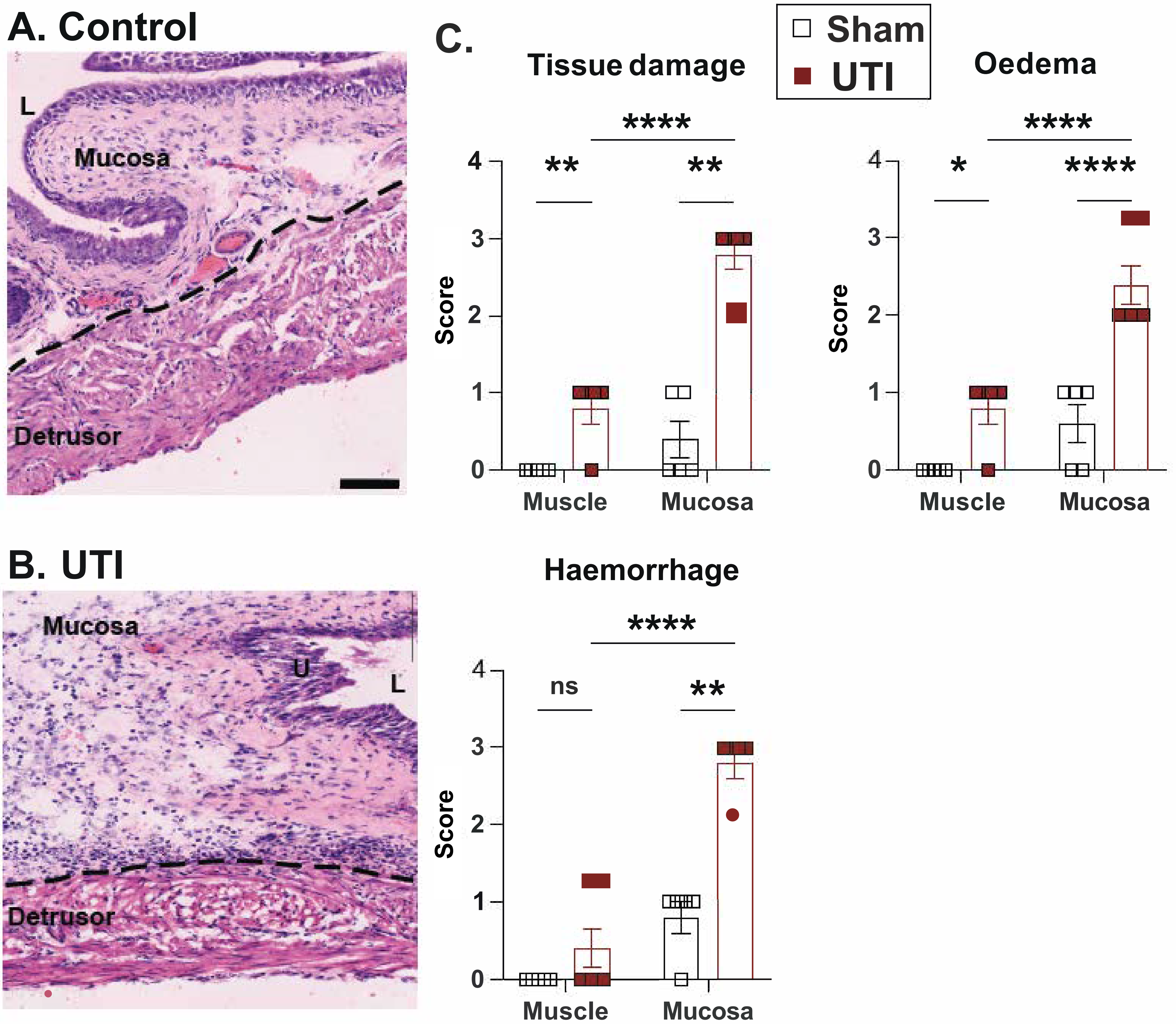

Figure S10. UPEC bladder infection evokes significant bladder damage which predominates in the mucosa.

H&E-stained bladder sections from mice 24hrs after sham (A) or UPEC instillation (B). (C) Histological quantification of bladder damage (using scoring criteria detailed in Table 2) demonstrates a significant increase in tissue damage, oedema, and haemorrhage, in bladders from UTI treated mice relative to Sham-treated mice. Scale bar=100μm. Data are presented as mean ± SEM and analysed by one-way ANOVA (n=5/group).

Supplementary Figure 11

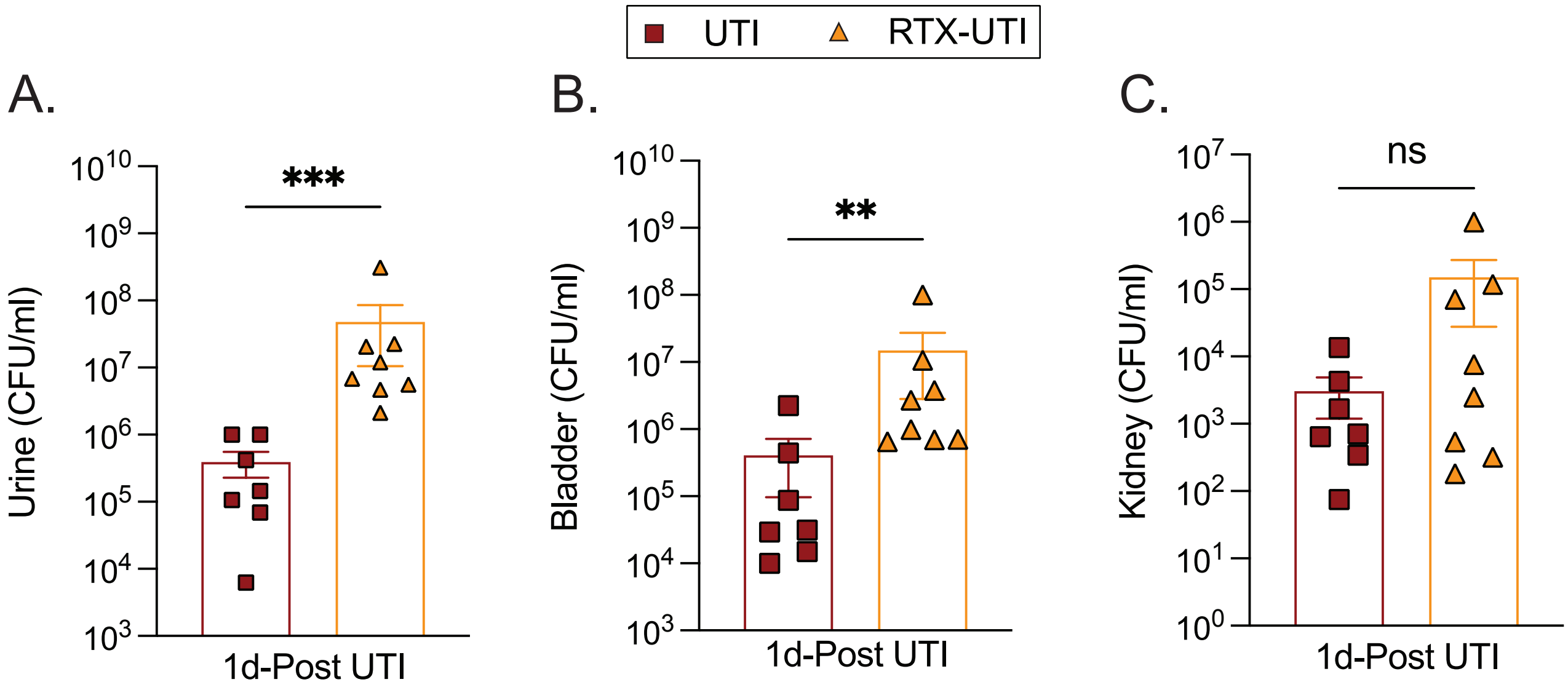

Figure S11. Bacterial load in urine, bladder, and kidneys following infection with UPEC strain UTI89 in sham and RTX-treated mice. Urine, bladders, and kidneys were isolated from mice 24hrs after infection. (**A**) Bacterial load in urine, bladder (**B**), and kidneys (**C**) 24hrs after mice were infected with UPEC strain UTI89 (N = 7-8 mice). Statistical differences were determined by unpaired t-test.

Figure S12

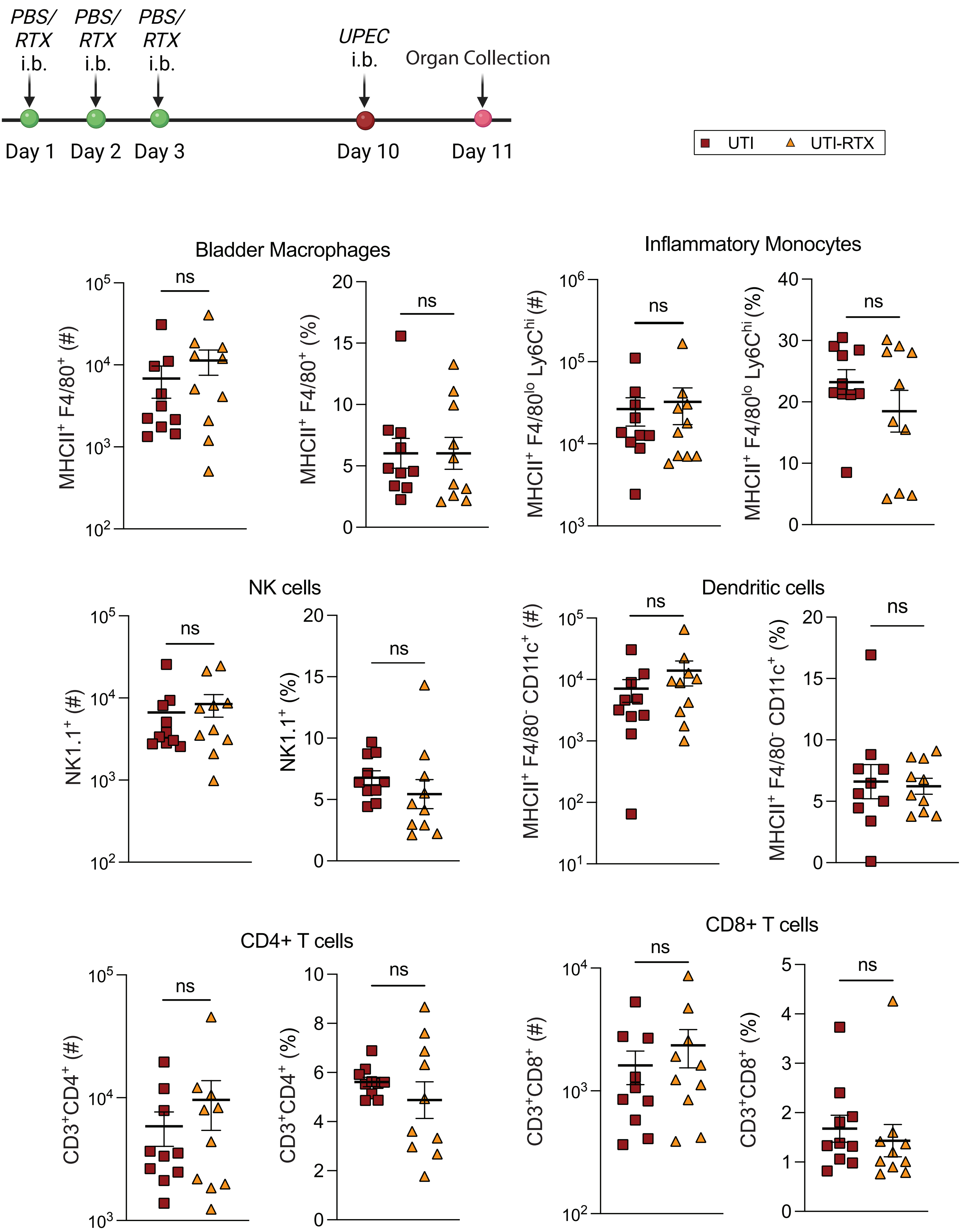

Figure S12. Quantification of immune cells from UTI and resiniferatoxin (RTX)-UTI bladders

Bladder immune cells were sorted and quantified using flow cytometry and the gating strategy outlined in Fig S12. Macrophages (MHCII$^+$ F4/80$^+$), Inflammatory monocytes (MHCII$^+$ F4/80^{lo} Ly6C^{hi}), NK cells (NK1.1$^+$), Dendritic cells (MHCII$^+$ F4/80$^-$ CD11c$^+$), CD4$^+$ T cells (CD3$^+$CD4$^+$) and CD8$^+$ T cells (CD3$^+$CD8$^+$), from bladders one day after UPEC instillation in mice pretreated with RTX (orange data points) compared to UPEC only (Red data points). Individual points represent cell counts (#) and cell frequency (%) from an individual mouse, N = 10 mice. Data was presented as mean ± SEM, Data were analysed by the non-parametric Mann-Whitney test for all datasets.

Figure S13

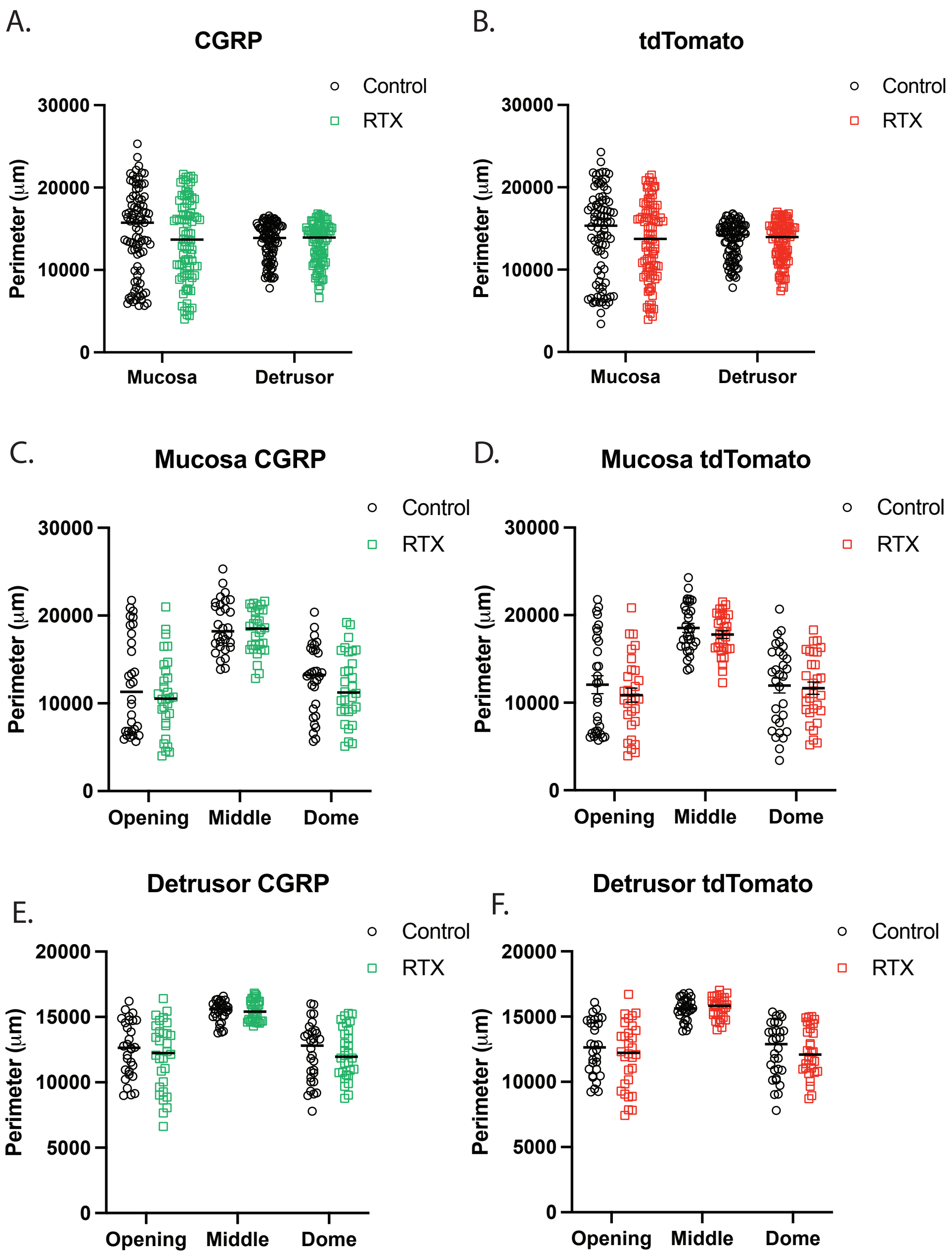

Figure S13 (in methods): Perimeter distance of control and RTX mucosa and detrusor bladder layers

Bladders from control and RTX-treated $Na_v1.8^{-Cre-tdTomato}$ mice were immunostained with CGRP were sectioned and imaged using the Zeiss Axio Scan.Z1 slide scanner. A total of n=90 sections from N=5 bladders/group were measured for perimeter of mucosa and detrusor using ImageJ. Individual measurements from the detrusor and mucosa were compared overall and at the opening, middle and dome of the bladder. This data was plotted to demonstrate the consistency in selecting areas within the detrusor and mucosa across all samples. The median and ranges was similar across all graphs: (A) CGRP overall, (B) tdTomato overall, (C) mucosa CGRP, (D) mucosa tdTomato, (E) detrusor CGRP and (F) detrusor tdTomato.

Figure S14

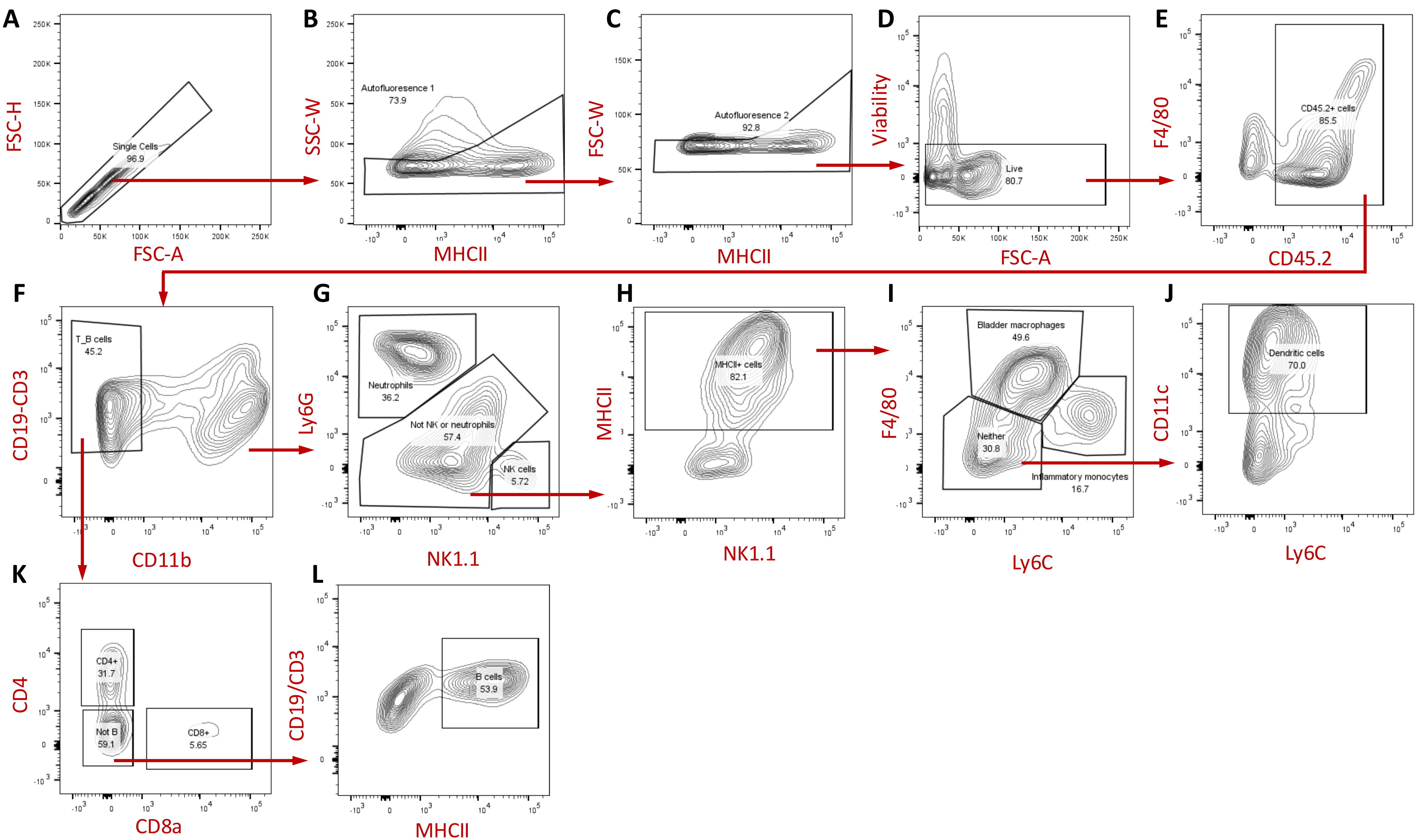

Figure S14 (In methods): Gating strategy for flow cytometry of immune cells in the bladder

(A) Forward scatter height (FSC-H) versus forward scatter area (FSC-A) plot is used to select single cells. (B) Side scatter width (SSC-W) versus MHCII to exclude autofluorescent cells, and further gating on MHCII+ cells is applied. (C) FSC-W versus MHCII for size exclusion of dead cells and debris. (D) Viability stain used to gate live cells. (E) Gating for CD45.2+ cells as a marker of immune cells. (F) CD19 and CD3 are used to gate out B cells (CD19+) and T cells (CD3+). CD11b is used to gate CD11b+ cells. (G) Ly6G and NK1.1 are used to distinguish neutrophils (Ly6G+), NK cells (NK1.1+), and other cell populations (Ly6G-/NK1.1-). (H) MHCII versus NK1.1 gating helps to further refine NK cells and other populations. (I) F4/80 and Ly6C are used to differentiate bladder macrophages (F4/80+), inflammatory monocytes (Ly6C+), and neutrophils. (J) CD11c and Ly6C gating identifies dendritic cells (CD11c+) and monocytes (Ly6C+). (K) CD4 versus CD8a is used to distinguish CD4+ helper T cells and CD8+ cytotoxic T cells among CD3+ populations. (L) MHCII versus CD19/CD3 shows MHCII+ cells with the CD19/CD3- population to identify antigen-presenting cells.
